# A generalized kinetic model for compartmentalization of organometallic catalysis†

**DOI:** 10.1039/d1sc04983f

**Published:** 2022-01-05

**Authors:** Brandon J. Jolly, Nathalie H. Co, Ashton R. Davis, Paula L. Diaconescu, Chong Liu

**Affiliations:** Department of Chemistry and Biochemistry, University of California Los Angeles California 90095 USA pld@chem.ucla.edu chongliu@chem.ucla.edu; California NanoSystems Institute (CNSI), University of California Los Angeles CA 90095 USA

## Abstract

Compartmentalization is an attractive approach to enhance catalytic activity by retaining reactive intermediates and mitigating deactivating pathways. Such a concept has been well explored in biochemical and more recently, organometallic catalysis to ensure high reaction turnovers with minimal side reactions. However, the scarcity of theoretical frameworks towards confined organometallic chemistry impedes broader utility for the implementation of compartmentalization. Herein, we report a general kinetic model and offer design guidance for a compartmentalized organometallic catalytic cycle. In comparison to a non-compartmentalized catalysis, compartmentalization is quantitatively shown to prevent the unwanted intermediate deactivation, boost the corresponding reaction efficiency (*γ*), and subsequently increase catalytic turnover frequency (TOF). The key parameter in the model is the volumetric diffusive conductance (*F*_V_) that describes catalysts' diffusion propensity across a compartment's boundary. Optimal values of *F*_V_ for a specific organometallic chemistry are needed to achieve maximal values of *γ* and TOF. As illustrated in specific reaction examples, our model suggests that a tailored compartment design, including the use of nanomaterials, is needed to suit a specific organometallic catalytic cycle. This work provides justification and design principles for further exploration into compartmentalizing organometallics to enhance catalytic performance. The conclusions from this work are generally applicable to other catalytic systems that need proper design guidance in confinement and compartmentalization.

## Abstract 2

Compartmentalization is an attractive approach to enhance catalytic activity by retaining reactive intermediates and mitigating deactivating pathways.

## Graphical abstract

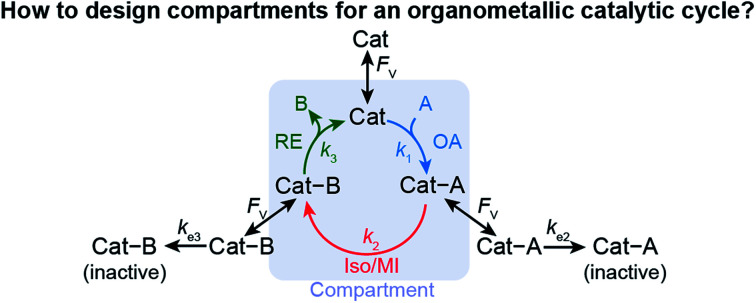

## Introduction

Compartmentalization has been well documented in biochemical literature as one method for achieving efficient *in vivo* tandem catalysis by encapsulating enzymes in well-defined micro- and nano-structures.^1–7^ By controlling the diffusion of species in and out of compartment boundaries, nature is able to retain reactive or toxic intermediates, increase local substrate concentration, and mitigate deactivating or competing pathways.^1–7^ For example, carboxysome microcompartments enhance the rate of CO_2_ fixation by encapsulating the cascade of carbonic anhydrase and ribose 1,5-bisphosphate carboxylase/oxygenase to generate high local concentration of CO_2_ and exclude deactivating O_2_ within their polyhedral structures.^8,9^ Also, the last two steps of tryptophan biosynthesis – the conversion of indole-3-glycerol-phosphate to indole and then to tryptophan – take advantage of the substrate-channeling effect bestowed by compartmentalized subunits of tryptophan synthase.^10,11^ Here, a hydrophobic tunnel between the two subunits retains the indole intermediate, which prevents its free diffusion and participation in deactivating side reactions.^10^ With billions of years of evolution, compartmentalization appears to be the mainstay of biology to manage the complex network of biochemical reactions that are frequently competing and incompatible with each other in a homogenous solution.

The success of natural compartmentalized enzyme cascades inspires the development of bio-mimetic synthetic catalysis with organometallic chemistry being the latest frontier. Multiple groups have employed well-defined spatial organization at the nano- and microscopic levels to construct *in vitro* biocatalytic and organometallic cascades with enhanced catalytic performance.^2,3,12–16^ Encapsulating NiFe hydrogenase in virus capsids improves its proteolytic and thermal stability as well as enhances the rate of H_2_ production.^12^ Confining a biochemical cascade of β-galactose, glucose oxidase, and horse radish peroxidase in metal–organic frameworks led to an enhancement of reaction yield in comparison to a freely diffusing analogue.^13,14^ The extent to which reaction yields are enhanced in confined enzyme cascades is reported to correlate with the distance between active sites, suggesting that spatial organization or localization of catalysts is beneficial in tandem or cascade reactions.^15^ In addition to biocatalysis, recently compartmentalization of organometallic catalysts has been experimentally demonstrated.^17–23^ For example, our group employed a nanowire-array electrode to pair seemingly incompatible CH_4_ activation based on O_2_-sensitive rhodium(ii) metalloradical (Rh(ii)) with O_2_-based oxidation for CH_3_OH formation.^17,24^ The application of a reducing potential to the nanowire array electrode created a steep O_2_ gradient within the wire array electrode, such that an anoxic compartment was established at the bottom of the wires. As a result that was not observable for planar electrode without an anoxic region, a catalytic cycle was formed in which the air-sensitive Rh(ii) activated CH_4_ in the O_2_-free region of the wire array electrode, while CH_3_OH synthesis proceeded in the aerobic domain with O_2_ as the terminal electron acceptor. The retainment of the ephemeral Rh(ii) intermediate by the nanowire electrode for catalytic CH_4_-to-CH_3_OH conversion^17,24^ encourages us to further explore the design principles of compartmentalizing cascades for higher turnovers with mitigated deactivation pathways.

**Fig. 1:**
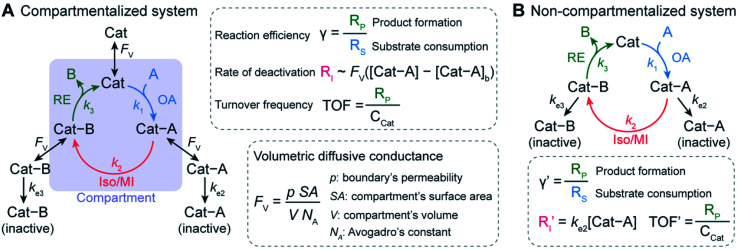
Schematic of a general compartmentalized catalytic cycle in organometallic chemistry (A) and the corresponding non-compartmentalized (freely diffusing) system (B). *γ*, reaction efficiency; *R*_S_, rate of substrate consumption; *R*_I_, rate of intermediate elimination; *R*_P_, rate of product formation; TOF, turnover frequency; *F*_V_, volumetric diffusive conductance; *p*, compartment boundary's permeability for catalytic intermediates; SA, compartment's surface area; *V*, compartment volume; *N*_A_, Avogadro's constant; OA, oxidative addition (rate constant *k*_1_); Iso/MI, isomerization/migratory insertion (rate constant *k*_2_) in conjunction with a competing deactivation (rate constant *k*_e2_); RE, reductive elimination (rate constant *k*_3_) in conjunction with a competing deactivation (rate constant *k*_e3_).

We envision that a theoretical framework for organometallic catalysis will expand the use of compartmentalization for organometallic chemistry and beyond. In biochemistry, mathematical modeling of confined enzyme cascades has been well developed and offers the design principles in natural systems^11,25^ and for engineered bio-compartments.^11,16,25,26^ The models pinpoint a key parameter, volumetric diffusive conductance (*F*_V_), which describes the diffusion propensity across a compartment's boundary. *F*_V_ is determined by a compartment's surface-to-volume ratio and its boundary's permeability.^26,27^ An optimal value of *F*_V_ tailored to the specific biochemical reactions are needed in order to achieve better reactivity in comparison to the non-compartmentalized alternative. Similarly, we contend that further development of compartmentalized organometallic chemistry demands a similar quantitative design principle. In a model organometallic cycle that includes oxidative addition (OA), isomerization/migratory insertion (Iso/MI), and reductive elimination (RE) along with undesirable deactivation pathways,^28^ what are the suitable values of the compartment's physical parameters for minimal deactivation and maximal turnover frequency (TOF) (Fig. 1)? Unfortunately, there has been a paucity of theoretical treatment for this question despite the exciting progresses in experimental demonstration.^17–23^ Such a lack of theoretical treatment motivates us to establish a general kinetic model and quantitatively investigate how compartmentalization will affect the competing reaction pathways and the corresponding turnover of the desired organometallic catalysis. The successful analysis of compartmentalization in organometallic catalysis, which bears the common features of catalysis in general, will pave the venue to analyze any catalytic cycle with synthetic compartments and confinement.

Here we report a general kinetic model and offer design guidance for a compartmentalized organometallic catalytic cycle. We took advantage of the established theoretical frameworks in biochemistry^16,25,26^ and applied such kinetic frameworks to a model compartmentalized cycle with competing deactivation pathways (Fig. 1A),^28^ and a non-compartmentalized counterpart as a control scenario (Fig. 1B). Under assumptions and simplifications applicable to organometallic catalysis, as a proof-of-concept we examined three important metrics of this catalytic cycle in both compartmentalized and non-compartmentalized scenarios: (1) reaction efficiency (*γ*) that gauges the percentage of intermediates funneled towards desirable catalytic turnover over deactivation pathways; (2) the deactivating rate of intermediate Cat − A(*R*_I_); and (3) the turnover frequency (TOF) that measures the steady-state catalytic rate despite intermediate deactivation. When compartments' *F*_V_ values are smaller than the intrinsic kinetics of the organometallic cycle in question, a compartmentalized system can significantly outperform a homogeneous counterpart with respect to *γ* and TOF with a lower value of *R*_I_. We illustrated the general relationship for specific organometallic catalysis to achieve maximal *γ* and TOF. We additionally employed the developed model to exemplarily analyze the experimental results and offer guidance of compartmentalization in nanowire-based CH_4_ activation,^17,29^ the Fujiwara–Mirotani reaction,^30,31^ and the Negishi coupling reaction.^32,33^ The established kinetic model can be adapted to suit a plethora of catalytic cycles with synthetic compartments, offering a framework to be expanded on for advanced compartmentalization of general chemical catalysis.

## Results and discussion

### Establishing a general kinetic framework of compartmentalization for an organometallic catalytic cycle

Our investigation starts with a hypothetical three-step organometallic cycle confined within a compartment in conjunction with multiple deactivation pathways in the exterior bulk solution (Fig. 1A and ESI Section 1A†).^28^ Catalytic species Cat of a presumed constant concentration in the bulk ([Cat]_*b*_≡*C*_Cat_) diffuses into the compartment of volume *V* and bind substrate molecule A through oxidative addition to form intermediate species Cat − A, either pseudo-first-order (*m* = 1)^34–37^ or pseudo-second-order (*m* = 2)^24,38,39^ with respect to Cat (rate constant *k*_1_). After a step of isomerization or migratory insertion (rate constant *k*_2_) converts Cat − A species to the product adduct Cat − B, the catalytic cycle is completed by the reductive elimination that transforms Cat − B back to Cat with the release of product B (rate constant *k*_3_). Here we presume that Cat, Cat − A and Cat − B intermediates all can diffuse across the compartment boundary and there are two possible competing deactivation pathways in the homogenous solution outside the compartment. The deactivations of Cat − A and Cat − B are presumed pseudo-first-order with respect to the intermediates with rate constants *k*_e2_ and *k*_e3_, respectively. Similarly, a non-compartmentalized system was constructed for the sake of comparison with the same set of kinetic reaction parameters (Fig. 1B and ESI Section 1B†). The established compartmentalized and non-compartmentalized catalytic cycles are generally applicable to a broad range of organometallic catalysis with concurrent deactivation processes,^28,40–42^ for which various deactivations have been well reviewed and comprehensively discussed in literature.^41^

For the compartmentalized scenario (Fig. 1A), we additionally assign volumetric diffusive conductance (*F*_V_) to quantitatively describe the extent of mass transport, predominantly diffusion-based, between the compartment and the surrounding bulk solution (ESI Section 2†). As a measure of molecules' propensities to diffusively cross the compartment's boundary under a given concentration gradient, *F*_V_ is defined as the product of compartment boundary's permeability (*ρ*) and its total surface area (SA) while divided by Avogadro's constant (*N*_A_) and the volume (*V*) of the corresponding compartment (Fig. 1A).^26^ In particular, *p* is proportional to the species' diffusion coefficients (*D*) and inversely proportional to the distance of diffusion path across the boundary.^43^ In our analysis, we assume *p* remains constant for Cat, Cat − A and Cat − B, given the fact that the catalytic center are frequently more bulky in comparison to the substrate/product, and the catalytic intermediates typically have similar diffusion coefficients despite the reaction-related adducts. We also assume that substrate A and product B are small enough that faster diffusion of A and B leads to minimal concentration gradients for A and B. Such assumption is also applicable to the practical applications when the substrates are used as the solvent in the catalysis that are pertinent to many organic/organometallic reactions. Under such assumptions, a single value of *F*_V_ for the catalytic intermediates is sufficient to describe the effect of compartmentalization on a catalytic cycle. Because the value of *ρ* depends on the compartment's physical properties, the design of compartment's surface-to-volume ratio (SA/*V*) and materials' properties at the compartment's boundary has significant impacts on the value of *F*_V_, and subsequently the overall catalytic turnover as will be discussed in this study.

In this work we aim to study the steady-state phenomena of compartmentalized catalysis. We assume constant, time-independent concentrations of Cat, Cat − A and Cat − B in both the compartment ([Cat], [Cat − A], and [Cat − B], respectively) as well as the surrounding bulk solution ([Cat]_*b*_≡*C*_Cat_ (*vide supra*), [Cat − A]_*b*_ and [Cat − B]_*b*_, respectively). Similarly, in the bulk solution substrate A is maintained at a constant concentration (*C*_A_) and fast removal of product B is ensured ([B] → 0). Such assumptions including [Cat]_*b*_≡*C*_Cat_ pertain to a flow reactor with sufficient amount of catalysts or a batch reaction under high catalyst loading and low conversion (ESI Section 1†). Alternatively, a constant total catalyst concentration including all catalytic species in the bulk can be presumed (*C*_Cat,total_≡[Cat]_*b*_ + [Cat − A]_*b*_ + [Cat − B]_*b*_ = constant), ESI Section 3†). We have analyzed the catalysis under both sets of assumptions. We note that the latter set of assumptions with a constant total catalyst concentration, more complicated to solve mathematically and labeled as “model *C*_Cat,total_” in ESI Section 3,† leads to similar conclusions and reinforces the general applicability of the following results solved when we assume [Cat]_*b*_≡*C*_cat_. Unless noted specifically, the results discussed below will be based on the former set of assumptions (ESI Section 1†).

A set of steady-state kinetic equations are constructed to reflect both the compartmentalized and non-compartmentalized scenarios (eqn (S1)–(S5) and (S67)–(S69)†) for an organometallic catalytic cycle following the analysis protocols established in biochemistry.^26^ Comparing to the non-compartmentalized case that only includes reactions in the homogenous solution (eqn (S67)–(S69)†), the equations for the compartmentalized case (eqn (S1)–(S5)†) additionally consider the reactions in the compartment as well as the mass transport across the boundary, whose magnitudes are governed by both the value of *F*_V_ and the concentration gradients across the compartment's boundary. Detailed mathematical treatment of the established equations can be found in ESI Section 1† and a few key outputs of the model are evaluated here. As one of the proposed benefits of compartmentalization is the capability of retaining reactive intermediates within the compartment without significant catalyst deactivation in the bulk,^2,3,14,16^ we are interested in evaluating the steady-state consumption rate of substrate A (*R*_S_), the generation rate of product B (*R*_P_), and the deactivation rate of intermediates Cat − A (*R*_I_) (Fig. 1A). Moreover, in both compartmentalized and non-compartmentalized scenarios, we aim to analyze the rate of reaction, numerically represented as turnover frequency TOF, and the efficacy of transforming the substrate A into targeted product B, numerically represented as reaction efficiency *γ* that is defined as the percentage of intermediates funneled towards desirable catalytic turnover.^16,26^ In both cases, *γ* is calculated as the ratio between the formation rate of product B and the consumption rate of substrate A. In the case of pseudo-first-order kinetics towards Cat in oxidative addition (*m* = 1), *γ*, *R*_I,*m*=1_, and TOF_*m*=1_ in a compartmentalized system can be expressed as,1
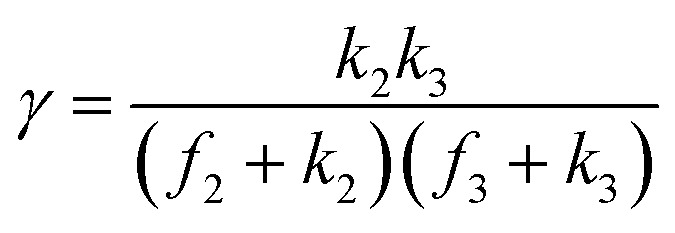
2
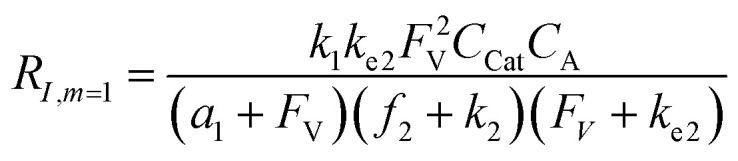
3
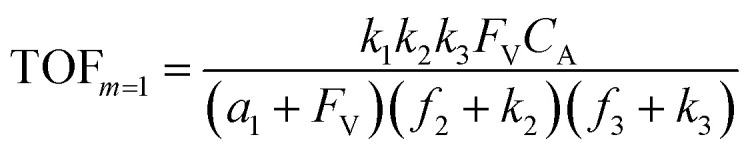
in which,4
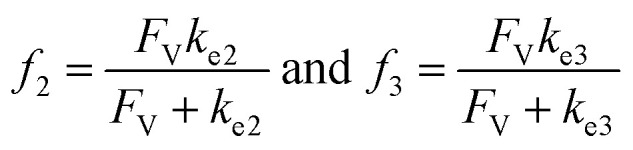
5
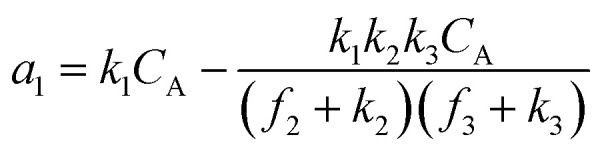


In comparison under a non-compartmentalized scenario, the corresponding *γ*′, 
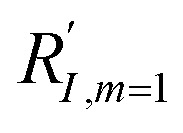
, and 
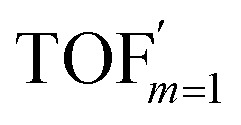
 are expressed as,6
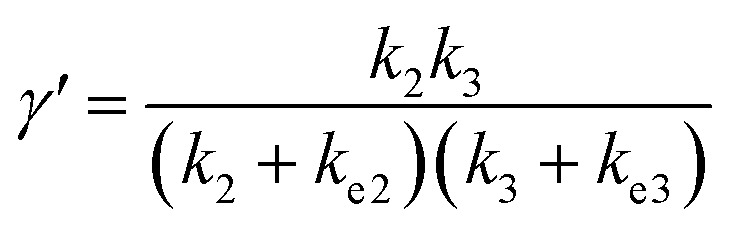
7
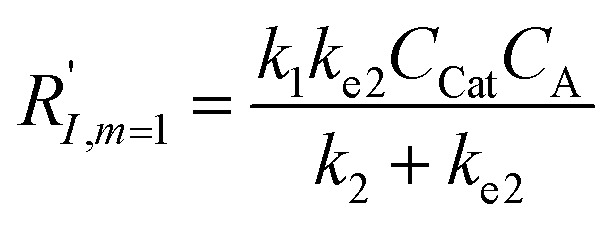
8
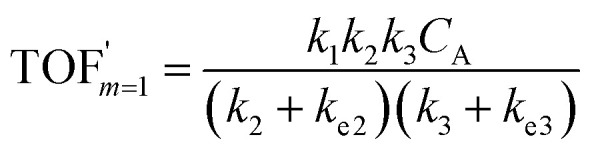


The mathematical expressions for *γ*, *R*_I_, and TOF results, derived from ESI Sections 1 and 3† for the assumptions outlined in the main text and for model *C*_Cat,total_ respectively, are summarized in Tables S1 and S2† as a reference. The successful construction and mathematical derivation of a general kinetic model in organometallic catalysis warrants quantitative evaluation about the efficacy of compartmentalization under different reaction kinetics and compartment properties.

### Exemplary numerical comparisons between compartmentalized and non-compartmentalized catalysis

The derived analytical solutions to the established kinetic model allow us to numerically calculate the values of *γ*, *R*_I_, and TOF in both compartmentalized and non-compartmentalized scenarios. Specifically, we set out to evaluate under what conditions compartmentalization is beneficial with a given set of parameters pertaining to the compartment's properties and kinetics of organometallic reactions. As an introductory example representative to a typical organometallic catalytic cycle, we assume that *C*_Cat_ = 1 mM and *C*_A_ = 10 mM, as organometallic catalytic systems often operate near 10 mol% catalyst loading.^40^ Values of kinetic parameters are *k*_1_ ∈ [10^−5^, 10^4^] M^−1^ s^−1^ (*m* = 1) (ref. 34, 35, 36 and 37) or *k*_1_ ∈ [10^−3^, 10^6^] M^−2^ s^−1^ (*m* = 2);^17,24,38,39^*k*_2_ ∈ [10^−3^, 10^6^] s^−1^(ref. 44 and 45) and *k*_3_ = 10^6^ s^−1^.^46,47^ When either *k*_1_ or *k*_2_ is not a variable of interest, they are set as *k*_1_ = 0.1 M^−1^ s^−1^ (*m* = 1) or 10 M^−2^ s^−1^ (*m* = 2) and *k*_2_ = 1 × 10^3^ s^−1^.^34–37^ The selection of those kinetic parameters is based on reviews of oxidative addition and migratory insertion, as well as reported kinetic studies using techniques such as time resolved infrared spectroscopy for transient species on the intermediates during carbonylation and O_2_ reduction and transfer, among others.^17,24,34–39,44,45^ The selection of *k*_3_ parameter value implicitly assumes fast reductive elimination from Cat − B, which is supported by the observation that reductive eliminations are often not the rate-determining step in a catalytic cycle.^46,47^ The values of deactivation kinetics *k*_e2_ for Cat − A and *k*_e3_ for Cat − B are selected with additional assumptions, given the dearth of reported kinetic values for the less exciting deactivation steps. As the reductive elimination from Cat − B is sufficiently fast, our primary focus is to examine the deactivation from Cat − A hence how the comparison between *k*_2_ and *k*_e2_ will affect the overall catalysis. Subsequently we assign *k*_e3_ = *k*_3_ = 1 × 10^6^ s^−1^ so that the rate of competing deactivation from Cat − B is no lower than rate of reductive elimination. Similarly, when *k*_e2_ is not a variable of interest, we set *k*_e2_ = *k*_2_ = 1 × 10^3^ s^−1^ to match the kinetics of isomerization/migratory insertion. Last, we set *F*_V_ ∈ [30, 600] s^−1^, whose range is estimated based on the diffusion coefficient of 9 × 10^−10^ m^2^ s^−1^ from tabulated organometallic catalysts,^48,49^ as well as the geometry and properties of reported microscopic compartments in porous materials, supramolecular assemblies, nanoscopic micelles, and the use of nanowire array electrode in our previous work (see ESI Section 2†).^17–20,29,50,51^ Overall, our selection of kinetic values here represents an organometallic catalytic cycle whose oxidative addition step is turnover-limiting and the deactivation of yielded Cat − A intermediate is the most critical issue, while the fast reductive elimination leaves the deactivation of Cat − B species secondary in terms of *γ* and TOF. With varying values of *F*_V_ and changing ratios between the values of *k*_2_ and *k*_e2_, the trend of compartmentalization's efficacy can be unveiled.

**Fig. 2:**
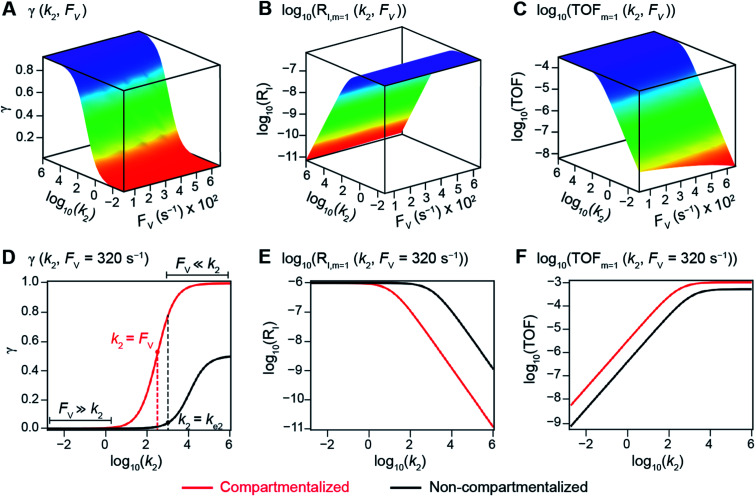
Reaction efficiency (*γ*, A and D), logarithmic of intermediate outflux rate (log_10_(*R*_I_), B and E), and logarithmic of turnover frequency (log_10_(TOF), C and F) as a function of volumetric diffusive conductance (*F*_V_) and logarithmic of the rate constant for isomerization or migratory insertion (Iso/MI) (log_10_(*k*_2_)). A to C, compartmentalized scenario depicted in Fig. 1A. D to F, comparisons between compartmentalized (red trace, when *F*_V_ = 320 s^−1^) and non-compartmentalized (black trace) scenarios. *m* = 1, *k*_1_ = 0.1 M^−1^ s^−1^ notwithstanding A and D, *k*_e2_ = 1 × 10^3^ s^−1^), *k*_3_ = *k*_e3_ = 1 × 10^6^ s^−1^. The selection of those exemplary values is based on literature reports on the kinetics of relevant organometallic systems (*vide supra*).

The numerically calculated values of *γ*, *R*_I_, and TOF as a function of *k*_2_ and *F*_V_ illustrate that compartmentalization generally outperforms the non-compartmentalized scenarios with a higher tolerance towards undesirable deactivation reactions (Fig. 2). Under a fixed rate constant of deactivation (*k*_e2_ = 1 × 10^3^ s^−1^) and pseudo-first-order oxidative addition (*k*_1_ = 0.1 M^−1^ s^−1^ for *m* = 1), values of *γ*, *R*_I_, and TOF in a compartmentalized system are plotted as a function of both *k*_2_ and *F*_V_ in Fig. 2A–C. The rate of isomerization/migratory insertion (*k*_2_) is understandably a predominant factor in all three plots. When *k*_2_ is much smaller than the rate of deactivation (*k*_e2_), *γ* approaches zero (Fig. 2A) when the deactivation of Cat − A outcompetes the step of isomerization/migratory insertion, which is concurrent with a higher rate of deactivation (*R*_I_ in Fig. 2B) and lower TOF value (Fig. 2C). Alternatively, when *k*_2_ is much larger than *k*_e2_ and the deactivation step is less relevant, *γ* plateaus towards unity with concomitant increase in TOF (Fig. 2A and C). Despite the dominant role of *k*_2_, whether or not the system is compartmentalized strongly affects the values of *γ*, *R*_I_, and TOF (Fig. 2D–F). While the trend is generally applicable for all values of *F*_V_, a specific case (*F*_V_ = 320 s^−1^) that corresponds to the nanowire array electrode for CH_4_-to-CH_3_OH conversion in our previous work,^17^ illustrates under which situation the advantages of compartmentalization will be observed. As the value of *k*_2_ increases, the compartmentalized scenario observes an increase of reaction efficiency *γ* in a sigmoidal fashion when *k*_2_ approaches the value of *F*_V_ (red trace in Fig. 2D); in contrast, *γ* in a non-compartmentalized case (black trace in Fig. 2D) won't increase until *k*_2_ approaches the value of *k*_e2_. Similarly, with *F*_V_ ≪ *k*_e2_ and under a reasonably large value of *k*_2_, compartmentalization suppresses the rate of deactivation *R*_I_ (Fig. 2E) and increases the TOF by roughly no less than one order of magnitude (Fig. 2F). Evaluations assuming pseudo-second-order kinetics towards Cat in the step of oxidative addition (*m* = 2) lead to the same conclusion (Fig. S3A and S4A†). Those observations suggest that the strategy of compartmentalization allows a catalytic cycle to be much more tolerant towards undesirable side reactions, as long as *F*_V_ is much smaller than *k*_e2_ (*F*_V_ ≪ *k*_e2_) with a judicious compartment design.

**Fig. 3:**
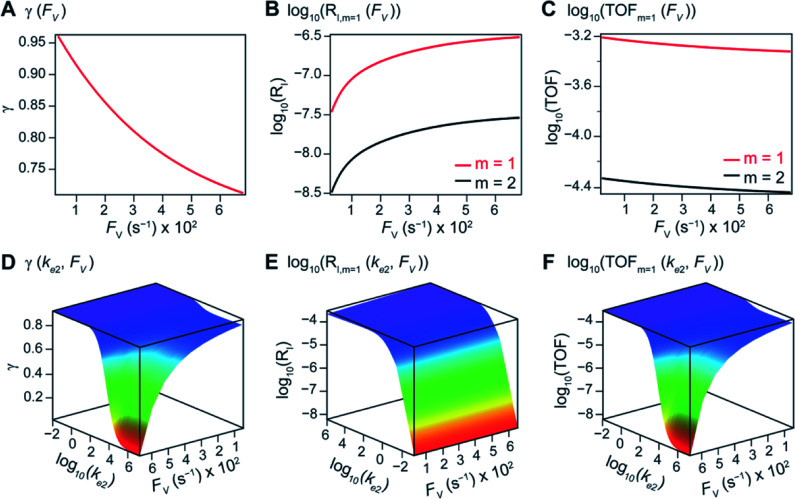
In a compartmentalized system, reaction efficiency (*γ*, A and D), logarithmic of intermediate outflux rate (log_10_(*R*_I_), B and E), and logarithmic of turnover frequency (log_10_(TOF), C and F) as a function of volumetric diffusive conductance (*F*_V_) and logarithmic of the rate constant for Cat − A deactivation (log_10_(*k*_e2_)). The axis of *F*_V_ and log_10_(*k*_e2_) in (D and F) are inverted for the sake of presentation clarity. *k*_1_ = 0.1 M^−1^ s^−1^ and 10 M^−2^ s^−1^ for *m* = 1 and 2, respectively, notwithstanding (A and D). *k*_2_ = *k*_e2_ = 1 × 10^3^ s^−1^. *k*_3_ = *k*_e3_ = 1 × 10^6^ s^−1^.

Additional examination suggests that a less “leaky” compartment, or one less prone to diffusive loss of intermediate, with smaller *F*_V_ value should be more effective than one with a relatively larger *F*_V_. Here the extent of leakiness is relevant to the reactions of interests and a “leaky” compartment is defined as one whose *F*_V_ is much larger than the one of *k*_2_ (*F*_V_ ≫ *k*_2_), with about one or two orders of magnitude of difference (a factor of 10 to 100) as shown in Fig. 2D, because the difference of *γ* values between compartmentalization and non-compartmentalization is the biggest when *F*_V_ < *k*_2_ ≪ *k*_e2_. Such a trend is more apparent when *γ*, *R*_I_, and TOF were plotted as a function of *F*_V_ under fixed values of *k*_2_ and *k*_e2_ (Fig. 3A–C). In both situations when *m* = 1 and *m* = 2, a larger value of *F*_V_ leads to smaller values of *γ* and TOF and large value of *R*_I_. This suggests that a more “leaky” compartment is not sufficient to conserve the yielded intermediates and is more prone to deactivation than one with a small *F*_V_. A similar conclusion can be obtained when investigating the dependence of *γ*, *R*_I_, and TOF as a function of *F*_V_ and *k*_e2_ (Fig. 3D–F, S3B and S4B†). Significant decrease of *γ* and increase of *R*_I_ was observed at high *F*_V_ values, particularly when the values of *k*_e2_ are so large that the deactivation is much faster than the isomerization/migratory insertion step and intermediate Cat − A has a much shorter life time once it diffuses out of the compartment.

The above noted observations can be mathematically rationalized from our derived equations. When the value of *F*_V_ is similar to or even larger than *k*_e2_ or *k*_e3_ (*F*_V_ ≳ *k*_e2_ or *k*_e3_),9
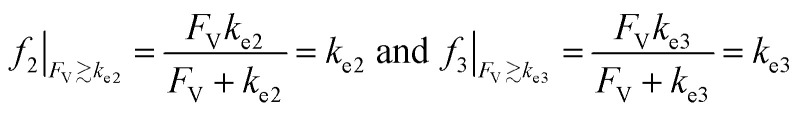


This will lead to *γ* ≈ *γ*′, *i.e.* the reaction efficiency is not significantly altered with compartmentalization in comparison to the non-compartmentalized case.

Alternatively, when *F*_V_ ≪ *k*_e2_ or *k*_e3_, we have10
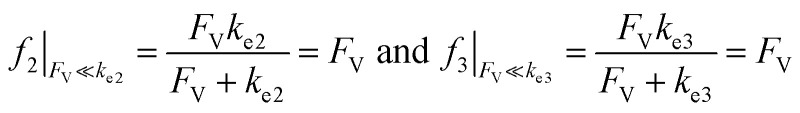


This leads to11
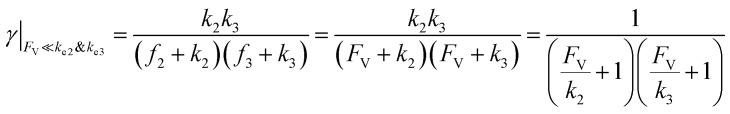
12
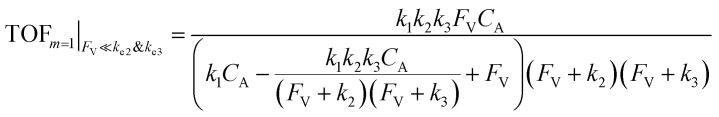


The equations noted above suggest that optimal, near-unity reaction efficiency *γ*, high TOF, and low *R*_I_ values would be obtained when *F*_V_ ≪ *k*_2_ and *k*_3_, which is consistent with our observations in Fig. 2. Under our above-stated assumption that isomerization/migratory insertion is the turnover-limiting step (*k*_2_ ≪ *k*_3_), *γ* = 0.9 and 0.99 when *F*_V_/*k*_2_ = 0.11 and 0.01, respectively. The corresponding expression of TOF can be simplified as,13
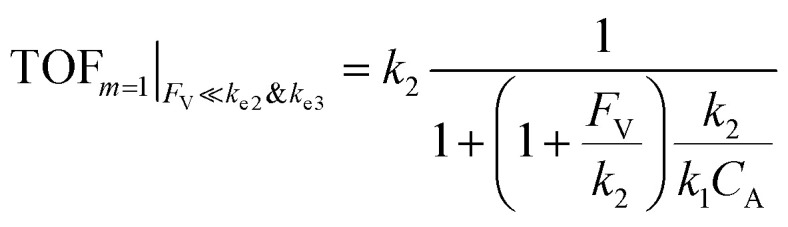
14
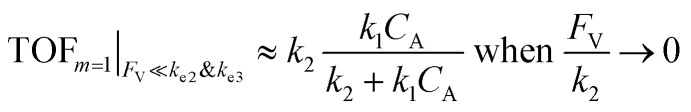


The monotonic yet asymptotic increase of TOF values suggests that maximal TOF will be achieved when *F*_V_/*k*_2_ → 0.

Lastly, when a constant total catalyst concentration including all catalytic species in the bulk is presumed (“model*C*_Cat,total_”, *C*_Cat,total_≡[Cat]_*b*_ + [Cat − A]_*b*_ + [Cat − B]_*b*_ = constant, ESI Section 3†), the calculated values of *R*_I_ and TOF as functions of *F*_V_ and *k*_2_ displayed little difference to the above-mentioned observations (Fig. S5–S7†), while the derived expressions of *γ* are identical under both assumptions (Tables S1 and S2†). Such observations suggest that slight variation in the assumptions of the developed model does not significantly alter how the compartment's *F*_V_ impacts the kinetics of overall catalysis.

### Implication on the design of compartmentalized catalysis

The established kinetic model and the numerical evaluation offers an affirmative answer to the efficacy of compartmentalized organometallic catalysis and, if needed, what is the desired properties of the established compartment. When the rate constants of the steps in the catalytic cycle (*k*_2_ and *k*_3_) are commensurate with or greater than the rate constants of deactivation steps (*k*_e2_ and *k*_e3_), *i.e. k*_2_ ≳ *k*_e2_ and *k*_3_ ≳ *k*_e3_, compartmentalization is not necessary since the intrinsic reactivity of catalysis is sufficiently fast with respect to undesirable side reactions. Compartmentalization should be considered under *k*_2_ < *k*_e2_ and *k*_3_ < *k*_e3_, when the intrinsic reactivities of the catalytic cycle cannot outcompete the deactivation pathways. The efficacy of compartmentalization will be observable, as long as the compartment's volumetric diffusive conductance *F*_V_ is much smaller than *k*_e2_ or *k*_e3_ (*F*_V_ ≪ *k*_e2_ or *k*_e3_). Nonetheless, one interesting conclusion from our analysis is that maximal efficacy of compartmentalization (reaction efficiency *γ* → 1) demands *F*_V_ to be smaller not only than the rate constants of deactivation steps (*k*_e2_ and *k*_e3_) but also than the rate constants of steps in the catalytic cycle (*k*_2_ and *k*_3_). This requirement for maximal *γ* stems from the fact that a “leaky” compartment with large *F*_V_ is not sufficient to conserve the yielded intermediates and is prone to deactivation. Practically, such a requirement is indeed a blessing for organometallic chemistry. As typical organometallic studies do not commonly characterize the deactivating side reactions, there lacks detailed kinetic information the values of *k*_e2_ or *k*_e3_ in comparison to the knowledge about catalytic kinetics (*k*_2_ and *k*_3_). Because we posit that criteria of *F*_V_ < *k*_2_ and *F*_V_ < *k*_3_ are sufficient for a compartment to “revive” a catalytic cycle unfunctional in a homogenous solution, kinetic information of the in-cycle steps (*k*_2_ and *k*_3_) is sufficient for future design of functional compartmentalization.

The feasibility of obtaining the range of *F*_V_ from the kinetics of the proposed catalytic cycle offers more guidance for the materials design for the compartment. As *F*_V_ is proportional to the compartment boundary's permeability (*ρ*) and its surface-to-volume ratio (SA/*V*),^26^ multiple synthetic handles could be applied to achieve a desirable *F*_V_ value. A less permeable interface at the boundary of compartment as well as smaller surface-to-volume ratio will help to reduce the mass transport hence the value of *F*_V_. Characterization techniques that help determine encapsulation geometry and assess permeability, such as electron microscopies and chromatographic methods, should be welcomed for more detailed mechanistic investigations in experimental demonstration.^52–55^ One interesting result from this argument is that a compartment of extremely small dimension, for example of nanoscopic scale, may not be necessarily beneficial, because nanoscopic dimensions with their large surface-to-volume ratio may create a “leaky” compartment. Here we set *F*_V_ ≫ *k*_2_ as the criterion for a “leaky” compartment that poorly retains intermediates (*vide supra*), when there is minimal difference in reaction efficiency *γ* between a compartmentalized and non-compartmentalized system (Fig. 2D). As *F*_V_ is calculated by a compartment's surface-to-volume ratio (SA/*V*), its boundary's permeability (*ρ*), and the Avogadro's constant (*N*_A_),^26^ a “leaky” compartment for a specific catalysis satisfies the following equations,15
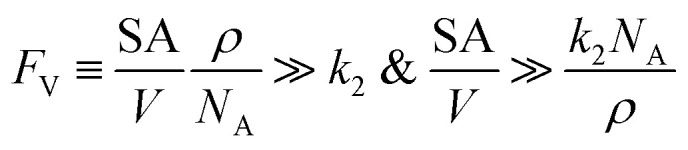


Provided a compartment's surface-to-volume ratio (SA/*V*) or chemicals' permeability across the compartment's boundary (*ρ*) is sufficiently large, our model predicts that compartmentalization will not be beneficial. In addition, the opposite inequality may be used to design optimal compartmentalized catalysis (*F*_V_ ≪ *k*_2_). Careful compartment design tailored to the specific kinetics of the catalytic cycle is recommended before experimental implementation.

The developed model remains generally applicable in the presence of mass transport heterogeneity in the compartment. As a first-order approximation, a mean-field average diffusion coefficient *D* is sufficient to describe the permeation of molecules through a compartment at ensemble level as long as the compartment's porosity is isotropic, based on single-molecule studies of molecular diffusion in mesoporous silica and polymer films.^56,57^ In the presence of anisotropicity such as highly aligned pores or in our previous work's nanowire arrays,^17,29^ a mean-field averaged diffusion coefficient *D* is still good enough to account for the diffusion phenomena in the specific direction.^58,59^ In cases where anisotropic diffusion exists, the values of anisotropic *ρ* normal to the compartment's boundary should be used when calculating *F*_V_. Moreover, in scenarios in where drastically heterogenous *D* values are apparent inside a single compartment, an effective value of *F̄*_V_ will be derived base on the volume-weighted average of *F*_V_ across the whole compartment, similar to the studies in metabolic microcompartments.^25^ The order of magnitudes of the derived *F̄*_V_ will be sufficient for the initial design of the compartmentalized catalysis, before further optimizations and detailed analysis proceed. Despite such additional mathematical treatment, how the *F*_V_ values are determined does not affect the validity and applicability of our development model in designing compartments.

We caution that our established model only considers the mass transport of catalysts and assumes an unconditionally fast supply of substrate A and quick removal of product B. While such assumptions have their real-life correspondence under certain circumstances (*vide supra*), the currently established model is incapable of accounting for the possible mass-transport limitation for the substrate and product, which could be induced by a small *F*_V_ value recommended by the model presented here. Given that, we cautioned that a lower bound of *F*_V_ exists for optimal performance in practical applications, and an unnecessarily small value of *F*_V_ could be detrimental to the compartment design. This argument is corroborated by our prior work that utilizes nanowire array electrode to pair CH_4_ activation from O_2_-sensitive metalloporphyrin with CH_3_OH generation with O_2_ as the terminal oxidant.^17,29^ An increase of the nanowire array's length, corresponding to a smaller value of *F*_V_ (ESI Section 2†), was experimentally observed to yield an increased rate of CH_4_ activation until the reaction rate plateaued for nanowire arrays of 27 μm length.^17,29^ Such experimental results illustrate the presence of a lower bound of *F*_V_ for optimal performance, when the mass transport of substrate CH_4_ is probably limited due to the increased length of the nanowire array.

### Examples to illustrate the utility of the developed model

We employed our model and analyzed the benefits of compartmentalizing the Fujiwara–Moritani reaction, which is a Pd-catalyzed oxidative C–C coupling reactions.^30,31^ In such a catalysis, stoichiometric oxidant is needed to regenerate the catalytically active Pd(ii) species,^30,31^ yet the presence of spent oxidant can inhibit the reaction and result in mediocre yields.^31,60^ Therefore, one possible strategy of reconciling such an incompatibility is to compartmentalize the Pd-based catalysts. Based on the available kinetic data reported in literature,^60^ we translated our generally applicable model into the Fujiwara–Moritani reaction (Fig. S8†) and established the mathematical relationship that correlates volumetric diffusive conductance (*F*_V_) with reaction efficiency (*γ*), the rate of catalyst deactivation (*R*_I_), and turnover frequency (TOF) (ESI Section 4†). We compared the values of *γ*, *R*_I_, and TOF between the compartmentalized and non-compartmentalized cases. While the homogenous non-compartmentalized scenario yields *γ* ∼ 10^−2^ and *R*_I_ ∼ 10^−5^ s^−1^, our model predicts that at *F*_V_ ∼ 10^−5^ s^−1^, about 100 times smaller than the kinetic constant *k*_2_ for the turnover-limiting step,^60^ compartmentalization significantly decreases the rate of deactivation (*R*_I_ = 2 × 10^−11^ s^−1^) and increase the reaction efficiency (*γ* = 0.99). Indeed, a recent experimental demonstration of compartmentalized Fujiwara–Moritani reaction with a “tube-in-tube” design is consistent with our model's prediction.^61^ As the minimally permeable tube-in-tube reactor is estimated to possess *F*_V_ ∼ 10^−28^ s^−1^ that well surpasses the criterion *F*_V_ ≪ *k*_2_ (*vide supra*), our model predicts little catalyst deactivation and high reaction efficiency (*γ* ∼ 1 and *R*_I_ ∼ 10^31^ s^−1^). Indeed, our predictions are consistent with the experimental observations^61^ and illustrate the utility of our model.

In another example, we analyzed the Negishi coupling reaction^32^ and concluded that compartmentalization of this reaction may have marginal benefits in the context of mitigating side reactions. In such a reaction, Ni or Pd catalysts enable the cross-coupling reactions between organic halides and organozinc, organoaluminum, or organozirconium compounds.^32,33^ Detailed kinetic information is available for the coupling between an aryl iodide compound with an aliphatic zinc chloride^33^ and we similarly established the kinetic model (Fig. S9 and ESI Section 5†). We found that in the non-compartmentalized scenario, *γ* is close to unity already (*γ* ∼ 1) because the deactivation steps (*k*_e2_ and *k*_e3_ ∼ 2 × 10^−3^ s^−1^) are slow in comparison to the steps in the catalytic cycle (*k*_1_ and *k*_2_ ∼ 1–10 M^−1^ s^−1^; *k*_3_ ∼ 0.5 s^−1^). This suggests that the benefits of compartmentalizing the Negishi coupling reaction will not be significant in the context of mitigating side reactions and boost reaction efficiency. Indeed, the model predicts that compartmentalization may even lower the TOF in comparison to the non-compartmentalized case, since the benefits of preventing already negligible side reactions is outweighed by the mass transport of catalyst. Overall, the developed model represents a viable tool to pick the catalytic reactions that are suitable for the study of compartmentalization.

## Conclusion

In this study, we developed a general kinetic framework for compartmentalizing organometallic catalysis with competing deactivation reactions in the bulk solution. Compartmentalization is only necessary under *k*_2_ < *k*_e2_ and *k*_3_ < *k*_e3_, when the intrinsic reactivities of the catalytic cycle cannot outcompete the deactivation pathways. Under such situations, the kinetic model predicts that careful compartment design with suitable values of volumetric diffusive conductance (*F*_V_ ≪ *k*_2_ and *F*_V_ ≪ *k*_3_ of at least one or two orders of magnitude difference) is capable of achieving maximal reaction efficiency (*γ*) and turnover frequency (TOF). Under our stated assumption that isomerization/migratory insertion is the turnover-limiting step (*k*_2_ ≪ *k*_3_), the criterion of minimal deactivation and maximal TOF is equivalent to *F*_V_/*k*_2_ = 0.11 and 0.01 for *γ* = 0.9 and 0.99, respectively, in order for the established compartment to minimize intermediate elimination and maximize catalysis. It is intriguing that the kinetics of deactivation steps are not needed for the design of compartment, as long as it is known that *k*_2_ < *k*_e2_ and *k*_3_ < *k*_*e*3_. As discussed with the examples of nanowire-based CH_4_ activation,^17,29^ Fujiwara–Mirotani reaction,^30,31^ and the Negishi coupling reaction,^32,33^ a tailored compartment design, including the use of nanomaterials, is needed to suit a specific organometallic catalysis. Such insights will assist in future *a priori* design of compartmentalized organometallics for enhanced catalytic performance. Moreover, the developed quantitative model is applicable to any general catalytic cycle particularly in the liquid phase, because the model includes the general features of any catalysis: multiple reaction steps connected in a cyclic fashion: the existence of turnover-limiting step, the interference from deactivation/competing reactions, and the issue of mass transport in the proximity of active sites. The conclusions and design principles obtained from the reported model is adaptable to suit most if not any catalytic cycles with synthetic compartments and confinements, offering a framework to be expanded on for advanced compartmentalization of general chemical catalysis.

## Author contributions

C. L. supervised the project. B. J. J. performed the mathematical derivations for the theoretical framework. N. H. C., A. R. D., and P. L. D. contributed to discussion about the Fujiwara–Mirotani and Negishi reactions. All authors discussed the results and assisted during the manuscript preparation.

## Conflicts of interest

The authors declare no competing financial interest.

## Supplementary Material

SC-013-D1SC04983F-s001
