# Supplementary material for: A generalized kinetic model for compartmentalization of organometallic catalysis
Source: Chem Sci. 2022 Jan 5;13(4):1101–10. doi: 10.1039/d1sc04983f (PMC8790775; doi:10.1039/d1sc04983f)
Supplement: SC-013-D1SC04983F-s001 [file SC-013-D1SC04983F-s001.pdf]

Electronic Supplementary Material (ESI) for Chemical Science.
This journal is © The Royal Society of Chemistry 2022

Supplementary Information

A Generalized Kinetic Model for Compartmentalization of Organometallic Catalysis

Brandon J. Jolly[1], Nathalie H. Co,[1] Ashton R. Davis,[1] Paula L. Diaconescu,[1*] Chong Liu[1,2*]

[1]Department of Chemistry and Biochemistry, University of California, Los Angeles, California 90095, United States

[2]California NanoSystems Institute (CNSI), University of California, Los Angeles, Los Angeles, CA 90095, USA

* To whom correspondence may be addressed. Email: pld@chem.ucla.edu, chongliu@chem.ucla.edu

Table of Contents

Methods *Page*

Section 1. Kinetic models for compartmentalized and non-compartmentalized scenarios with assumptions stated in the main text S4

Section 1A. Mathematical derivation of compartmentalized scenarios S4

Section 1B. Mathematical derivation of non-compartmentalized scenarios S9

Section 2. Derivation of F_V for a nanowire array as a nanoscopic compartment in solution S12

Section 3. Kinetic models for compartmentalized and non-compartmentalized scenarios for model $C_{Cat,total}$ S14

Section 3A. Mathematical derivation of compartmentalized scenarios for model $C_{Cat,total}$ S14

Section 3B. Mathematical derivation of non-compartmentalized scenarios for model $C_{Cat,total}$ S17

Section 4. Calculation of γ, TOF, and R_I for tube-in-tube Fujiwara-Mirotani reaction S19

Section 5. Calculation of γ, TOF, and R_I for the Negishi reaction S21

Supplementary Tables

Table S1. Expressions for compartmentalized and non-compartmentalized key reaction metrics S23

Table S2. Expressions for compartmentalized and non-compartmentalized key reaction metrics for model $C_{Cat,total}$ S24

Supplementary Figures

Figure S1. Compartmentalized rate of intermediate outflux (R_I) as a function of F_V and k_1 with first and second order (m = 1, 2) on initial catalyst concentration ($[Cat]$) S25

Figure S2. Compartmentalized TOF as a function of F_V and k_1 for m = 1 and m = 2 S26

Figure S3. Compartmentalized R_I as a function of F_V and k_2 or k_{e2} for m = 2 S27

Figure S4. Compartmentalized TOF as a function of F_V and k_2 or k_{e2} for m = 2 S28

Figure S5. Compartmentalized R_I and TOF as functions of F_V and k_2 for m = 1 under model $C_{Cat,total}$ S29

Figure S6. Compartmentalized R_I and TOF as a function of F_V for m = 1 under model $C_{Cat,total}$ S30

Figure S7. Comparison between compartmentalized and non−compartmentalized R_I and TOF as a function of k_2 for m = 1 under model $C_{Cat,total}$ S31

Figure S8. Catalytic cycle for Fujiwari-Mirotani palladium catalyzed olefin arylation operating in a tube-in-tube reactor S32

Figure S9. Catalytic cycle for Negishi palladium catalyzed cross couplings S33

Additional references S34

Section 1. Kinetic models for compartmentalized and non-compartmentalized scenarios with assumptions stated in the main text

The steps of a chosen general catalytic cycle are shown in Figure 1A and are outlined in the main text. Here, C_{Cat} represents the concentration of the initial catalyst in bulk. C_{Cat} is assumed to be present in excess of $[Cat]$, which represents the initial catalyst concentration in the compartment. Therefore, C_{Cat} is assumed to negligibly change during the course of the cascade catalysis, and it is thus treated as a constant.

1A. Mathematical derivation of compartmentalized scenarios

Here, we solve for key reaction metrics for a compartmentalized catalytic cycle under the assumptions stated in the first section of the results and discussion of the main text. The steady state kinetic equations for catalytic species in the compartmentalized system are:

$$\frac{d[Cat]}{dt} = F_V(C_{Cat} - [Cat]) + k_3[Cat - B] - k_1[Cat]^m C_A = 0 \quad \text{(S1)}$$

$$\frac{d[Cat - A]}{dt} = -F_V([Cat - A] - [Cat - A]_b) + k_1[Cat]^m C_A - k_2[Cat - A] = 0 \quad \text{(S2)}$$

$$\frac{d[Cat - A]_b}{dt} = F_V([Cat - A] - [Cat - A]_b) - k_{e2}[Cat - A]_b = 0 \quad \text{(S3)}$$

$$\frac{d[Cat - B]}{dt} = -F_V([Cat - B] - [Cat - B]_b) + k_2[Cat - A] - k_3[Cat - B] = 0 \quad \text{(S4)}$$

$$\frac{d[Cat - B]_b}{dt} = F_V([Cat - B] - [Cat - B]_b) - k_{e3}[Cat - B]_b = 0 \quad \text{(S5)}$$

Expressions for F_V and $k_{e2/3}$ are shown in Section S3. From Equation S5, $[Cat - B]_b$ may be solved for:

$$F_V[Cat - B] - F_V[Cat - B]_b - k_{e3}[Cat - B]_b = 0 \quad \text{(S6)}$$

$$F_V[Cat - B] = F_V[Cat - B]_b + k_{e3}[Cat - B]_b \quad \text{(S7)}$$

$$[Cat - B]_b(F_V + k_{e3}) = F_V[Cat - B] \quad \text{(S8)}$$

$$[Cat - B]_b = \frac{F_V[Cat - B]}{F_V + k_{e3}} \tag{S9}$$

Inputting Equation S9 into Equation S4, $[Cat - B]$ may be solved for:

$$-F_V[Cat - B] + F_V[Cat - B]_b + k_2[Cat - A] - k_3[Cat - B] = 0 \tag{S10}$$

$$k_2[Cat - A] = F_V[Cat - B] - F_V[Cat - B]_b + k_3[Cat - B] \tag{S11}$$

$$k_2[Cat - A] = F_V[Cat - B] - F_V\left(\frac{F_V[Cat - B]}{F_V + k_{e3}}\right) + k_3[Cat - B] \tag{S12}$$

$$k_2[Cat - A] = [Cat - B]\left(F_V - \frac{F_V^2}{F_V + k_{e3}} + k_3\right) \tag{S13}$$

$$[Cat - B] = \frac{k_2[Cat - A]}{\frac{F_V k_{e3}}{F_V + k_{e3}} + k_3} \tag{S14}$$

$$f_n = \frac{F_V k_{en}}{F_V + k_{en}} \tag{S15}$$

$$[Cat - B] = \frac{k_2[Cat - A]}{f_3 + k_3} \tag{S16}$$

From Equation S3, $[Cat - A]_b$ may be solved for:

$$F_V([Cat - A] - [Cat - A]_b) - k_{e2}[Cat - A]_b = 0 \tag{S17}$$

$$F_V[Cat - A] - F_V[Cat - A]_b - k_{e2}[Cat - A]_b = 0 \tag{S18}$$

$$F_V[Cat - A] = [Cat - A]_b(F_V + k_{e2}) \tag{S19}$$

$$[Cat - A]_b = \frac{F_V[Cat - A]}{F_V + k_{e2}} \tag{S20}$$

Inputting Equation S20 into Equation S2, $[Cat - A]$ may be solved for:

$$-F_V([Cat - A] - [Cat - A]_b) + k_1[Cat]^m[A] - k_2[Cat - A] = 0 \tag{S21}$$

$$k_1[Cat]^m C_A = F_V[Cat-A] - F_V[Cat-A]_b + k_2[Cat-A] \tag{S22}$$

$$k_1[Cat]^m C_A = F_V[Cat-A] - F_V\left(\frac{F_V[Cat-A]}{F_V + k_{e2}}\right) + k_2[Cat-A] \tag{S23}$$

$$[Cat-A] = \frac{k_1[Cat]^m C_A}{F_V - \frac{F_V^2}{F_V + k_{e2}} + k_2} \tag{S24}$$

$$[Cat-A] = \frac{k_1[Cat]^m C_A}{f_2 + k_2} \tag{S25}$$

Rearranging S1 to solve for $[Cat]$ when m = 1, 2

$$F_V(C_{Cat} - [Cat]) + k_3[Cat-B] - k_1[Cat]^m C_A = 0 \tag{S26}$$

$$k_1[Cat]^m C_A + F_V[Cat] - F_V C_{Cat} - k_3[Cat-B] = 0 \tag{S27}$$

Input solution for $[Cat-B]$ (S16) and $[Cat-A]$ (S25)

$$k_1[Cat]^m C_A + F_V[Cat] - F_V C_{Cat} - k_3\left(\frac{k_2[Cat-A]}{f_3 + k_3}\right) = 0 \tag{S28}$$

$$k_1[Cat]^m C_A + F_V[Cat] - F_V C_{Cat} - \frac{k_1 k_2 k_3 [Cat]^m C_A}{(f_2 + k_2)(f_3 + k_3)} = 0 \tag{S29}$$

$$\left(k_1 C_A - \frac{k_1 k_2 k_3 C_A}{(f_2 + k_2)(f_3 + k_3)}\right)[Cat]^m + F_V[Cat] - F_V C_{Cat} = 0 \tag{S30}$$

$$defining\ a_1 = k_1 C_A - \frac{k_1 k_2 k_3 C_A}{(f_2 + k_2)(f_3 + k_3)} \tag{S31}$$

When m = 1

$$a_1[Cat] + F_V[Cat] - F_V C_{Cat} = 0 \tag{S32}$$

$$(a_1 + F_V)[Cat] = F_V C_{Cat} \tag{S33}$$

$$[Cat] = \frac{F_V C_{Cat}}{a_1 + F_V} \tag{S34}$$

When m = 2

$$a_1[Cat]^2 + F_V[Cat] - F_V C_{Cat} = 0 \quad (S35)$$

$$[Cat] = \frac{-F_V \pm \sqrt{F_V^2 - 4(a_1)(-F_V C_{Cat})}}{2a_1} \quad (S36)$$

$$[Cat] = \frac{-F_V + \sqrt{F_V^2 + 4a_1 F_V C_{Cat}}}{2a_1} \quad (S37)$$

Key Reaction Metrics: Compartmentalized

$$R_{S,m} = k_1[Cat]^m C_A \quad (S38)$$

$$R_{I,m} = F_V([Cat-A] - [Cat-A]_b) \quad (S39)$$

$$R_{P,m} = k_3[Cat-B] \quad (S40)$$

$$\gamma_m = \frac{R_{P,m}}{R_{S,m}} \quad (S41)$$

$$TOF_m = \frac{R_{P,m}}{mC_{Cat}} \quad (S42)$$

Solving for R_S when m = 1 by inputting S34 into S38

$$R_{S,m=1} = k_1[Cat]C_A \quad (S43)$$

$$R_{S,m=1} = \frac{k_1 F_V C_{Cat} C_A}{a_1 + F_V} \quad (S44)$$

Solving for R_S when m = 2 by inputting S37 into S38

$$R_{S,m=2} = k_1[Cat]^2 C_A \quad (S45)$$

$$R_{S,m=2} = k_1 \left(\frac{-F_V + \sqrt{F_V^2 + 4a_1 F_V C_{Cat}}}{2a_1} \right)^2 C_A \quad (S46)$$

Simplifying R_I (S39)

$$R_{I,m} = F_V \left(\frac{k_1[Cat]^m C_A}{f_2 + k_2} - \frac{F_V \left(\frac{k_1[Cat]^m C_A}{f_2 + k_2} \right)}{F_V + k_{e2}} \right) \tag{S47}$$

$$R_{I,m} = F_V k_1 [Cat]^m C_A \left(\frac{1}{f_2 + k_2} - \frac{F_V}{(f_2 + k_2)(F_V + k_{e2})} \right) \tag{S48}$$

$$R_{I,m} = \frac{k_1 k'_{e2} F_V [Cat]^m C_A}{(f_2 + k_2)(F_V + k_{e2})} \tag{S49}$$

Solving for R_I when m = 1 by inputting S34 into S49

$$R_{I,m=1} = \left(\frac{k_1 k_{e2} F_V C_A}{(f_2 + k_2)(F_V + k_{e2})} \right) [Cat] \tag{S50}$$

$$R_{I,m=1} = \frac{k_1 k_{e2} F_V^2 C_{Cat} C_A}{(a_1 + F_V)(f_2 + k_2)(F_V + k_{e2})} \tag{S51}$$

Solving for R_I when m = 2 by inputting S37 into S49

$$R_{I,m=2} = \left(\frac{k_1 k_{e2} F_V C_A}{(f_2 + k_2)(F_V + k_{e2})} \right) [Cat]^2 \tag{S52}$$

$$R_{I,m=2} = \frac{k_1 k_{e2} F_V C_A}{(f_2 + k_2)(F_V + k_{e2})} \left(\frac{-F_V + \sqrt{F_V^2 + 4a_1 F_V C_{Cat}}}{2a_1} \right)^2 \tag{S53}$$

Simplifying R_P (S40)

$$R_{P,m} = k_3 [Cat - B] \tag{S54}$$

$$R_{P,m} = \frac{k_1 k_2 k_3 [Cat]^m C_A}{(f_2 + k_2)(f_3 + k_3)} \tag{S55}$$

Solving for R_P when m = 1 by inputting S34 into S55

$$R_{P,m=1} = \frac{k_1 k_2 k_3 C_A}{(f_2 + k_2)(f_3 + k_3)} [Cat] \tag{S56}$$

$$R_{P,m=1} = \frac{k_1 k_2 k_3 F_V C_{Cat} C_A}{(a_1 + F_V)(f_2 + k_2)(f_3 + k_3)} \tag{S57}$$

Solving for R_P when m = 2 by inputting S37 into S55

$$R_{P,m=2} = \frac{k_1 k_2 k_3 C_A}{(f_2 + k_2)(f_3 + k_3)} [Cat]^2 \tag{S59}$$

$$R_{P,m=2} = \frac{k_1 k_2 k_3 C_A}{(f_2 + k_2)(f_3 + k_3)} \left(\frac{-F_V + \sqrt{F_V^2 + 4a_1 F_V C_{Cat}}}{2a_1} \right)^2 \tag{S60}$$

Solving for γ (S41)

$$\gamma_m = \frac{k_3 [Cat - B]}{k_1 [Cat]^m [A]} \tag{S61}$$

$$\gamma_m = \frac{k_3 \left(\frac{k_2 [Cat - A]}{f_3 + k_3} \right)}{k_1 [Cat]^m [A]} \tag{S62}$$

$$\gamma_m = \frac{k_2 k_3 \left(\frac{k_1 [Cat]^m C_A}{f_2 + k_2} \right)}{k_1 [Cat]^m [A] (f_3 + k_3)} \tag{S63}$$

$$\gamma = \frac{k_2 k_3}{(f_2 + k_2)(f_3 + k_3)} \tag{S64}$$

Solving for TOF_m when m = 1, 2 by inputting S57 or S60 into S42

$$TOF_{m=1} = \frac{k_1 k_2 k_3 F_V C_A}{(a_1 + F_V)(f_2 + k_2)(f_3 + k_3)} \tag{S65}$$

$$TOF_{m=2} = \frac{k_1 k_2 k_3 C_A}{2C_{Cat}(f_2 + k_2)(f_3 + k_3)} \left(\frac{-F_V + \sqrt{F_V^2 + 4a_1 F_V C_{Cat}}}{2a_1} \right)^2 \tag{S66}$$

1B. Mathematical derivation of non-compartmentalized scenarios

The rates of species within the general catalytic cycle under the non-compartmentalized framework are generated by dropping diffusive (F_V) terms and bulk concentrations from the compartmentalized framework to generate the following under steady state:

$$\frac{d[Cat]}{dt} = k_3 [Cat - B] - k_1 [Cat]^m C_A = 0 \tag{S67}$$

$$\frac{d[Cat - A]}{dt} = k_1 [Cat]^m C_A - k_2 [Cat - A] - k_{e2} [Cat - A] = 0 \tag{S68}$$

$$\frac{d[Cat-B]}{dt} = k_2[Cat-A] - k_3[Cat-B] - k_{e3}[Cat-B] = 0 \tag{S69}$$

Solving for $[Cat-B]$ from S69

$$k_2[Cat-A] - k_3[Cat-B] - k_{e3}[Cat-B] = 0 \tag{S70}$$

$$[Cat-B](k_3 + k_{e3}) = k_2[Cat-A] \tag{S71}$$

$$[Cat-B] = \frac{k_2[Cat-A]}{k_3 + k_{e3}} \tag{S72}$$

Solving for $[Cat-A]$ from S68

$$k_1[Cat]^m C_A - k_2[Cat-A] - k_{e2}[Cat-A] = 0 \tag{S73}$$

$$[Cat-A](k_2 + k_{e2}) = k_1[Cat]^m C_A \tag{S74}$$

$$[Cat-A] = \frac{k_1[Cat]^m C_A}{k_2 + k_{e2}} \tag{S75}$$

Key Reaction Metrics: Non-Compartmentalized

$$R_{S,m} = k_1[Cat]^m C_A \tag{S38}$$

$$R_{I,m} = k_{e2}[Cat-A] \tag{S76}$$

$$R_{P,m} = k_3[Cat-B] \tag{S40}$$

$$\gamma_m = \frac{R_{P,m}}{R_{S,m}} \tag{S41}$$

$$TOF_m = \frac{R_{P,m}}{m[Cat]} \tag{S42}$$

Solving for R_I where m = 1, 2 by inputting S75 into S76

$$R_I = k_{e2}[Cat-A] \tag{S77}$$

$$R_I = k_{e2}\left(\frac{k_1[Cat]^m C_A}{k_2 + k_{e2}}\right) \tag{S78}$$

$$R_I = \frac{k_1 k_{e2}[Cat]^m C_A}{k_2 + k_{e2}} \quad with\, m = 1, 2 \tag{S79}$$

Solving for $R_{P3,m}$ where m = 1, 2 by inputting S72 into S40

$$R_{P,m} = k_3[Cat - B] \tag{S80}$$

$$R_{P,m} = k_3\left(\frac{k_1 k_2[Cat]^m C_A}{(k_2 + k_{e2})(k_3 + k_{e3})}\right) \tag{S81}$$

$$R_{P,m} = \frac{k_1 k_2[Cat]^m C_A}{(k_2 + k_{e2})(k_3 + k_{e3})} \quad m = 1, 2 \tag{S82}$$

Solving for γ_m

$$\gamma_m = \frac{k_3[Cat - B]}{k_1[Cat]^m C_A} \tag{S83}$$

$$\gamma_m = \frac{\frac{k_1 k_2[Cat]^m C_A}{(k_2 + k_{e2})(k_3 + k_{e3})}}{k_1[Cat]^m C_A} \tag{S84}$$

$$\gamma = \frac{k_2 k_3}{(k_2 + k_{e2})(k_3 + k_{e3})} \tag{S85}$$

Solving for TOF_m where m = 1, 2 by inputting S82 into S42

$$TOF_{m=1} = \frac{k_1 k_2 k_3 C_A}{(k_2 + k_{e2})(k_3 + k_{e3})} \tag{S86}$$

$$TOF_{m=2} = \frac{k_1 k_2 k_3[Cat] C_A}{2(k_2 + k_{e2})(k_3 + k_{e3})} \tag{S87}$$

To mirror the assumption made in the prior compartmentalized scenario (section S1A) that C_{Cat} is present in excess of compartmentalized $[Cat]$, here we assume the extent of $[Cat]$ consumption in a non-compartmentalized cycle with deactivations is minimal relative to its regeneration, therefore $[Cat] \approx C_{Cat}$ = 1 mM. Limitations associated with this assumption are addressed in section S3 by introducing a $C_{Cat,total}$ term and are shown to yield negligible difference in mechanistic conclusions.

Section 2. Derivation of F_V for a nanowire array as a nanoscopic compartment in solution

We expand on our previous derivation of term $F_V = F/VN_A$, where F is diffusive conductance in M s^{-1}, V is either the volume of the compartment or the bulk (denoted V_b), and N_A is Avogadro's number.[1,2] In brief, F is defined as the product of compartment permeability (ρ), surface area (SA), and Avogadro's number (N_A). We define permeability in terms diffusion coefficient (D), diffusion path to enter the compartment (Δx)[3], and nanowire length (L) as follows:

$$\rho \approx \frac{D}{\Delta x} \tag{S88}$$

We approximate that the compartment is established roughly halfway down the wire based on our prior work, therefore $\Delta x \sim 0.5 \times L$.[4] Combining p with SA and N_A, we obtain:

$$F \approx \frac{D * SA * N_A}{0.5 * L} \tag{S89}$$

In order to obtain the flux of particular species, we normalize F to $V \times N_A$, which we define as F_V. We derive V in terms of $0.5 \times SA$ and length of the compartment along the nanowire, $L - \Delta x = 0.5 \times L$, to obtain:

$$F_V = \frac{F}{VN_A} \approx \frac{\frac{D \times SA \times N_A}{0.5 * L}}{0.5 \times SA \times 0.5 \times L \times N_A} \approx \frac{8D}{L^2} \tag{S90}$$

We make a first order approximation where substrate and product molecules A and B are relatively small, therefore D is not significantly changed throughout the course of the cycle and we assume all catalytic species have the same D and flux governed by the same F_V value.[4] In the example of a nanowire array, F_V is only a function of nanowire length L. We suspect that this approximation may be extended to other processes to tune F_V solely based on nanostructure geometry. However, we note one limitation of this derivation of F_V is that it assumes equal probability for a molecule to diffuse in and out of the compartment at every nanowire length. Our approach to reconcile this issue is to take an integral weighted average of F_V at each nanowire length. Therefore, we use the following expression to explicitly calculate F_V at various L, ranging from 10 – 50 μm based on previous experimental work.[4]

$$\frac{\int_L^0 F_V(L) w(L) dL}{\int_L^0 w(L)\, dL} \tag{S91}$$

To account for bulk volume (V_b) in deactivation steps, we introduce k'_{en} terms for the compartmentalized system, using $Cat - A$ as an example.

$$\frac{d[Cat-A]}{dt} = \frac{F}{V_b N_A}([Cat-A]-[Cat-A]_b) - k_{e2}[Cat-A]_b = 0 \quad (S92)$$

$$\frac{d[Cat-A]}{dt} = \frac{F}{V N_A}([Cat-A]-[Cat-A]_b) - k_{e2}\frac{V_b}{V}[Cat-A]_b = 0 \quad (S93)$$

$$\frac{d[Cat-A]}{dt} = F_V([Cat-A]-[Cat-A]_b) - k'_{e2}[Cat-A]_b = 0 \quad (S94)$$

$$k'_{en} = k_{en}\frac{V_b}{V} \quad (S95)$$

By using k'_{en} (n = 2, 3), we allow the previous F_V equation with the volume of the compartment and subsequent calculations to be utilized throughout the model. In addition, we make a similar approximation that $k'_{en} \approx k_n$ for eliminations to be considered. For simplicity, only k_{en} is listed in the main text and throughout the derivations, however for compartmentalized systems, k'_{en} should be used.

Section 3. Kinetic models for compartmentalized and non-compartmentalized scenarios for model $C_{Cat,total}$

We note the assumption that initial catalyst concentration in the bulk ($[Cat]_b$) does not change over time and that C_{Cat} may be used as a constant may not always hold true. Here in this scenario, we introduce $[Cat]_b$ and $C_{Cat,total}$ terms to avoid treating catalyst concentration as a constant.

3A. Mathematical derivation of compartmentalized scenarios for model $C_{Cat,total}$

Equations S1 – S5 are the same, however we introduce eq S88 to account for $[Cat]_b$ and $C_{Cat,total}$.

$$\frac{d[Cat]}{dt} = -F_V([Cat] - [Cat]_b) + k_3[Cat - B] - k_1[Cat]^m C_A = 0 \tag{S1}$$

$$\frac{d[Cat - A]}{dt} = -F_V([Cat - A] - [Cat - A]_b) + k_1[Cat]^m C_A - k_2[Cat - A] = 0 \tag{S2}$$

$$\frac{d[Cat - A]_b}{dt} = F_V([Cat - A] - [Cat - A]_b) - k_{e2}[Cat - A]_b = 0 \tag{S3}$$

$$\frac{d[Cat - B]}{dt} = -F_V([Cat - B] - [Cat - B]_b) + k_2[Cat - A] - k_3[Cat - B] = 0 \tag{S4}$$

$$\frac{d[Cat - B]_b}{dt} = F_V([Cat - B] - [Cat - B]_b) - k_{e3}[Cat - B]_b = 0 \tag{S5}$$

$$C_{Cat,total} = [Cat] + [Cat]_b + [Cat - A] + [Cat - A]_b + [Cat - B] + [Cat - B]_b \tag{S96}$$

Expressions for $[Cat - A]$ (S25), $[Cat - A]_b$ (S20), $[Cat - B]$ (S16), and $[Cat - B]_b$ (S9) are unchanged from the scenario without $C_{Cat,total}$. Now solving for $[Cat]_b$ from S96:

$$[Cat]_b = \frac{C_{Cat,total}}{[Cat] + [Cat - A] + [Cat - A]_b + [Cat - B] + [Cat - B]_b} \tag{S97}$$

$$[Cat]_b = \frac{C_{Cat,total}}{[Cat] + \left(\frac{k_1[Cat]^m C_A}{f_2 + k_2}\right) + \left(\frac{F_V[Cat - A]}{F_V + k_{e2}}\right) + \left(\frac{k_1k_2k_3[Cat]^m C_A}{(f_2 + k_2)(f_3 + k_3)}\right) + \frac{F_V[Cat - B]}{F_V + k_{e3}}} \tag{S98}$$

$$[Cat]_b = \frac{C_{Cat,total}}{[Cat] + \frac{k_1[Cat]^m C_A}{f_2 + k_2} + \frac{k_1F_V[Cat]^m C_A}{(f_2 + k_2)(F_V + k_{e2})} + \frac{k_1k_2k_3[Cat]^m C_A}{(f_2 + k_2)(f_3 + k_3)} + \frac{k_1k_2k_3F_V[Cat]^m C_A}{(f_2 + k_2)(f_3 + k_3)(F_V + k_{e3})}} \tag{S99}$$

Solving for $[Cat]_b$ when m = 1:

$$[Cat]_{b,m=1} = \frac{C_{Cat,total}}{[Cat]\left(1 + \frac{k_1 C_A}{f_2 + k_2} + \frac{k_1 F_V C_A}{(f_2 + k_2)(F_V + k_{e2})} + \frac{k_1 k_2 k_3 C_A}{(f_2 + k_2)(f_3 + k_3)} + \frac{k_1 k_2 k_3 F_V C_A}{(f_2 + k_2)(f_3 + k_3)(F_V + k_{e3})}\right)} \quad \text{(S100)}$$

$$a_2 = 1 + \frac{k_1 C_A}{f_2 + k_2} + \frac{k_1 F_V C_A}{(f_2 + k_2)(F_V + k_{e2})} + \frac{k_1 k_2 k_3 C_A}{(f_2 + k_2)(f_3 + k_3)} + \frac{k_1 k_2 k_3 F_V C_A}{(f_2 + k_2)(f_3 + k_3)(F_V + k_{e3})} \quad \text{(S101)}$$

$$[Cat]_b = \frac{C_{Cat,total}}{a_2}[Cat]^{-1} \quad \text{(S102)}$$

Solving for $[Cat]$ when m = 1:

$$-F_V([Cat] - [Cat]_b) + k_3[Cat - B] - k_1[Cat]C_A = 0 \quad \text{(S103)}$$

$$-F_V[Cat] + F_V\left(\frac{C_{Cat,total}}{a_2}[Cat]^{-1}\right) + \frac{k_1 k_2 k_3 [Cat] C_A}{(f_2 + k_2)(f_3 + f_3)} - k_1[Cat]C_A = 0 \quad \text{(S104)}$$

$$-F_V[Cat] + F_V\left(\frac{C_{Cat,total}}{a_2}[Cat]^{-1}\right) + a_1[Cat] = 0 \quad \text{(S105)}$$

$$-F_V[Cat]^2 + \frac{F_V C_{Cat,total}}{a_2} + a_1[Cat]^2 = 0 \quad \text{(S106)}$$

$$[Cat]^2(F_V - a_1) = \frac{F_V C_{Cat,total}}{a_2} \quad \text{(S107)}$$

$$[Cat] = \sqrt{\frac{F_V C_{Cat,total}}{a_2(F_V - a_1)}} \quad \text{(S108)}$$

Solving for $[Cat]_b$ when m = 2:

$$[Cat]_{b,m=2} = \frac{C_{Cat,total}}{[Cat] + \frac{k_1[Cat]^2 C_A}{f_2 + k_2} + \frac{k_1 F_V [Cat]^2 C_A}{(f_2 + k_2)(F_V + k_{e2})} + \frac{k_1 k_2 k_3 [Cat]^2 C_A}{(f_2 + k_2)(f_3 + f_3)} + \frac{k_1 k_2 k_3 F_V [Cat]^2 C_A}{(f_2 + k_2)(f_3 + f_3)(F_V + k_{e3})}} \quad \text{(S109)}$$

$$[Cat]_{b,m=2} = \frac{C_{Cat,total}}{[Cat] + [Cat]^2\left(\frac{k_1 C_A}{f_2 + k_2} + \frac{k_1 F_V C_A}{(f_2 + k_2)(F_V + k_{e2})} + \frac{k_1 k_2 k_3 C_A}{(f_2 + k_2)(f_3 + f_3)} + \frac{k_1 k_2 k_3 F_V C_A}{(f_2 + k_2)(f_3 + f_3)(F_V + k_{e3})}\right)} \quad \text{(S110)}$$

$$a_3 = \frac{k_1 C_A}{f_2 + k_2} + \frac{k_1 F_V C_A}{(f_2 + k_2)(F_V + k_{e2})} + \frac{k_1 k_2 k_3 C_A}{(f_2 + k_2)(f_3 + f_3)} + \frac{k_1 k_2 k_3 F_V C_A}{(f_2 + k_2)(f_3 + f_3)(F_V + k_{e3})} \quad \text{(S111)}$$

$$[Cat]_{b,m=2} = C_{Cat,total}([Cat] + a_3[Cat]^2)^{-1} \quad \text{(S112)}$$

Solving for $[Cat]$ when m = 2:

$$-F_V([Cat]-[Cat]_b)+k_3[Cat-B]-k_1[Cat]C_A=0 \tag{S113}$$

$$-F_V[Cat]+F_V\left(C_{Cat,total}([Cat]+a_3[Cat]^2)^{-1}\right)+\frac{k_1k_2k_3[Cat]C_A}{(f_2+k_2)(f_3+f_3)}-k_1[Cat]C_A=0 \tag{S114}$$

$$-F_V[Cat]+F_V\left(C_{Cat,total}([Cat]+a_3[Cat]^2)^{-1}\right)+a_1[Cat]=0 \tag{S115}$$

$$-F_V[Cat]([Cat]+a_3[Cat]^2)+F_VC_{Cat,total}+a_1[Cat]([Cat]+a_3[Cat]^2)=0 \tag{S116}$$

$$-F_V[Cat]^2-F_Va_3[Cat]^3+F_VC_{Cat,total}+a_1[Cat]^2+a_3[Cat]^3=0 \tag{S117}$$

$$(a_3-F_V)[Cat]^3+(a_1-F_V)[Cat]^2+F_VC_{Cat,total}=0 \tag{S118}$$

Defining $b=(a_3-F_V)$, $c=(a_1-F_V)$, $d=F_VC_{Cat,total}$, a general solution is:

$$[Cat]=\frac{1}{3}\left(\frac{\sqrt[3]{-27b^2d+3\sqrt{3}\sqrt{27b^4d^2+4b^2c^3d}-2c^3}}{\sqrt[3]{2}b}+\frac{\sqrt[3]{2}c^2}{b\sqrt[3]{-27b^2d+3\sqrt{3}\sqrt{27b^4d^2+4b^2c^3d}-2c^3}}-\frac{c}{b}\right) \tag{S119}$$

Key Reaction Metrics: Compartmentalized

$$R_{I,m}=F_V([Cat-A]-[Cat-A]_b) \tag{S39}$$

$$\gamma_m=\frac{R_{P,m}}{R_{S,m}} \tag{S41}$$

$$TOF_m=\frac{R_{P,m}}{m[Cat]_0} \tag{S42}$$

γ does not change from when accounting for $C_{Cat,total}$ as $[Cat-A]$ and $[Cat-B]$ do not change, leaving the solution previously obtained where γ does not depend on $[Cat]$ (S61-64).

Solving for R_I when m = 1:

$$R_{I,m}=F_V([Cat-A]-[Cat-A]_b) \tag{S120}$$

Using prior simplification of R_I (S49) since $[Cat-A]$ and $[Cat-A]_b$ do not change:

$$R_{I,m}=\frac{k_1k_{e2}F_V[Cat]^mC_A}{(f_2+k_2)(F_V+k_{e2})} \tag{S121}$$

$$R_{I,m=1} = \frac{k_1 k_{e2} F_V C_A}{(f_2 + k_2)(F_V + k_{e2})} \sqrt{\frac{F_V C_{Cat,total}}{a_2 (F_V - a_1)}} \quad \text{(S122)}$$

Solving for TOF when m = 1:

$$TOF_m = \frac{R_{P,m}}{m C_{Cat,total}} \quad \text{(S42)}$$

$$TOF_{m=1} = \frac{k_1 k_2 k_3 [Cat] C_A}{C_{Cat,total}(f_2 + k_2)(f_3 + f_3)} \quad \text{(S123)}$$

$$TOF_{m=1} = \frac{k_1 k_2 k_3 C_A}{(f_2 + k_2)(f_3 + f_3)} \sqrt{\frac{F_V}{C_{Cat,total} a_2 (F_V - a_1)}} \quad \text{(S124)}$$

3B. Mathematical derivation of non-compartmentalized scenarios for model $C_{Cat,total}$

Here, we derive an analogous non-compartmentalized framework under model $C_{Cat,total}$ to compare to the non-compartmentalized scenario derived in section S1B with the original assumptions from the main text. Eq S67 – 69 remain the same, however we introduce eq S125 to solve for $[Cat]$ in terms of $C_{Cat,total}$. We note that again γ is unchanged from Section 1 when accounting for $C_{Cat,total}$ (S64).

$$C_{Cat,total} = [Cat] + [Cat - A] + [Cat - B] \quad \text{(S125)}$$

Inputting expressions for $[Cat - A]$ (S75) and $[Cat - B]$ (S72), which are unchanged when accounting for $C_{Cat,total}$.

$$C_{Cat,total} = [Cat] + \frac{k_1 [Cat] C_A}{k_2 + k_{e2}} + \frac{k_1 k_2 [Cat] C_A}{(k_2 + k_{e2})(k_3 + k_{e3})} \quad \text{(S126)}$$

$$C_{Cat,total} = [Cat] \left(1 + \frac{k_1 C_A}{k_2 + k_{e2}} + \frac{k_1 k_2 C_A}{(k_2 + k_{e2})(k_3 + k_{e3})}\right) \quad \text{(S127)}$$

$$a_4 = 1 + \frac{k_1 C_A}{k_2 + k_{e2}} + \frac{k_1 k_2 C_A}{(k_2 + k_{e2})(k_3 + k_{e3})} \quad \text{(S128)}$$

$$[Cat] = \frac{C_{Cat,total}}{a_4} \tag{S129}$$

The non-compartmentalized simplification of R_I (S79) is unchanged, therefore $R_{I,m=1}$ under this scenario can be derived as the following:

$$R_{I,m=1} = \frac{k_1 k_{e2} C_{Cat,total} C_A}{a_4(k_2 + k_{e2})} \tag{S130}$$

Solving for $TOF_{m=1}$ starting from S42:

$$TOF_{m=1} = \frac{k_1 k_2 k_3 C_A}{a_4(k_2 + k_{e2})(k_3 + k_{e3})} \tag{S131}$$

Section 4. Calculation of γ, TOF, and R_I for tube-in-tube Fujiwara-Mirotani reaction.

In the confines of the kinetic model developed in this study and established mechanistic understanding of Pd catalyzed olefin arylation (Fujiwara-Mirotani),[5-7] shown schematically in Figure S8 operating in the tube-in-tube reactor,[8] we arrive at the below steady state compartmentalized expressions. (S132 – S136). We assign the Pd(II) species as Pd, the amide carbonyl coordinated intermediate species as $Pd-A$, and the aryl bound species as $Pd-B$, analogous to the generic Cat, $Cat-A$, and $Cat-B$ species utilized earlier. C_{aryl} refers to the concentration of the aryl substrate, and C_{olefin} represents the concentration of the olefin substrate.

$$\frac{d[Pd]}{dt} = F_V(C_{Pd} - [Pd]) + k_3[Pd-B]C_{olefin} - k_1[Pd]^m C_{aryl} = 0 \tag{S132}$$

$$\frac{d[Pd-A]}{dt} = -F_V([Pd-A] - [Pd-A]_b) + k_1[Pd]^m C_{aryl} - k_2[Pd-A] = 0 \tag{S133}$$

$$\frac{d[Pd-A]_b}{dt} = F_V([Pd-A] - [Pd-A]_b) - k_{e2}[Pd-A]_b = 0 \tag{S134}$$

$$\frac{d[Pd-B]}{dt} = -F_V([Pd-B] - [Pd-B]_b) + k_2[Pd-A] - k_3[Pd-B]C_{olefin} = 0 \tag{S135}$$

$$\frac{d[Pd-B]_b}{dt} = F_V([Pd-B] - [Pd-B]_b) - k_{e3}[Pd-B]_b = 0 \tag{S136}$$

Below are the resultant expressions for compartmentalized γ, R_I, and TOF, with γ being unchanged from S64, and with R_I and TOF containing the appropriate palladium, aryl and olefin substrate concentration terms.

$$\gamma = \frac{k_2 k_3 C_{olefin}}{(f_2 + k_2)(f_3 + k_3 C_{olfein})} \tag{S137}$$

$$R_I = \left(\frac{k_1 k_{e2} F_V C_{aryl}}{(f_2 + k_2)(F_V + k_{e2})}\right)[Pd_{cat}] \tag{S138}$$

$$TOF = \frac{k_1 k_2 k_3 F_V C_{aryl} C_{olefin}}{(a_1 + F_V)(f_2 + k_2)\left(f_3 + k_3 C_{olefin}\right)} \tag{S139}$$

The expressions for non-compartmentalized γ', $R_I{}'$, and TOF' are as follows.

$$\gamma' = \frac{k_2 k_3 C_{olefin}}{(k_2 + k_{e2})(k_3 C_{olefin} + k_{e3})} \tag{S140}$$

$$R_I' = \frac{k_1 k_{e2} [Pd_{cat}] C_{aryl}}{k_2 + k_{e2}} \tag{S141}$$

$$TOF' = \frac{k_1 k_2 k_3 C_{aryl} C_{olefin}}{(k_2 + k_{e2})(k_3 C_{olefin} + k_{e3})} \tag{S142}$$

An approximate F_V was estimated based the tube-in-tube reactor as follows:

$$F_V = \frac{\left(\frac{D}{\Delta \mathrm{x}}\right) SA}{VN_A} \tag{S143}$$

Here, surface area (SA), volume (V), and diffusion path (Δx) are calculated from reported dimensions of the reactor, taken to be cylindrical. Similar to our treatment of a nanowire array generated compartment (Supplementary Information Section 2), an average diffusion path of half of the diameter is utilized. Using a representative 2 m long reactor with a radius of 1 mm, as well as an approximate diffusion coefficient (D) on the order of magnitude of 10^{-10} m^{-2} s^{-1} (see also Supplementary Information Section 5 for common Pd catalysts diffusion coefficient), F_V is estimated to be 10^{-28} s^{-1}. This is corroborated by Pd leaching studies,[8] which also lead to $F_V \sim 10^{-28}$ s^{-1} This approximate value was determined by converting a typical leaching rate[8] of 0.1 ppm hr^{-1} to diffusive conductance (F in M s^{-1}), and finally to volumetric diffusive conductance (F_V, in s^{-1}) using $F_V = F/VN_A$.

Kinetic data from was compiled from prior reports,[6,7] with $k_1 \sim 2$ M^{-1} s^{-1}, $k_2 \sim 3\times10^{-3}$ s^{-1}, $k_3 \sim 0.15$ M^{-1} s^{-1}, $k_{e2} \sim 3\times10^{-5}$ s^{-1}, and $k_{e3} \sim 5$ s^{-1}. Concentrations were set to 1×10^{-6}, 0.1, and 0.25 M for the Pd catalyst, aryl and olefin substrate respectively, following literature reports at low catalyst loading.[7,8] Taking the above into consideration, we obtain $\gamma \sim 1$, $R_I \sim 3\times10^{-31}$ s^{-1}, and $TOF \sim 0.5$ s^{-1} for the compartmentalized Fujiwara-Mirotani reaction. The non-compartmentalized treatment in comparison leads to $\gamma \sim 8\times10^{-3}$, $R_I \sim 7\times10^{-5}$ s^{-1}, and $TOF \sim 5\times10^{-3}$ s^{-1}. Such an analysis confirms on a theoretical basis the benefits of compartmentalization to this system over a homogeneous analogue and demonstrates the validity of our model. Though we note reported TOF for the tube-in-tube reactor is lower (0.005^{-1}),[8] suggesting our analysis is an overestimate of true TOF.

Section 5. Calculation of γ, TOF, and R$_I$ for the Negishi reaction.

In this section, we analyze palladium catalyze cross coupling (Negishi reaction),[9,10] as a hypothetical compartmentalized system and non-compartmentalized analogue. We arrive at the below equations following its catalytic cycle, shown schematically in Figure S9.

$$\frac{d[Pd]}{dt} = F_V(C_{Pd} - [Pd]) + k_3[Pd - B] - k_1[Pd]^m C_{ArI} = 0 \quad \text{(S144)}$$

$$\frac{d[Pd - A]}{dt} = -F_V([Pd - A] - [Pd - A]_b) + k_1[Pd]^m C_{ArI} - k_2[Pd - A]C_{CyZnCl} = 0 \quad \text{(S145)}$$

$$\frac{d[Pd - A]_b}{dt} = F_V([Pd - A] - [Pd - A]_b) - k_{e2}[Pd - A]_b = 0 \quad \text{(S146)}$$

$$\frac{d[Pd - B]}{dt} = -F_V([Pd - B] - [Pd - B]_b) + k_2[Pd - A]C_{CyZnCl} - k_3[Pd - B] = 0 \quad \text{(S147)}$$

$$\frac{d[Pd - B]_b}{dt} = F_V([Pd - B] - [Pd - B]_b) - k_{e3}[Pd - B]_b = 0 \quad \text{(S148)}$$

Here, the initial catalyst species, $[Pd]$ (typically $Pd(PR_3)X_2$) is labelled as Pd, the aryl iodide bound species post oxidative addition is labelled $Pd - A$, and the aryl cyclohexyl bound species pre reductive elimination is labelled $Pd - B$, with the substrate taken as the aryl iodide (ArI). For the compartmentalized system, we chose to confine this reaction within the metal organic framework (MOF) with formula $\{Cu_6Sr[(S,S)\text{-Mecysmox}]_3(OH)_2(H_2O)\}15H_2O$ (Mecysmox = bis[S-methylcysteine]oxalyl diamide), because it is well characterized and has been shown to stabilize a Pd catalyst within its pores.[11] We have averaged the diffusion coefficient based on previous studies on Pd complexes as 6×10^{-10} m^2•s^{-1}.[12] An F_V value for this system was approximated accounting for geometric constraints of the MOF compartments[12] using the definition of F_V displayed in Figure 1 and discussed in Supplementary Information Section 2 (eq S143).

$$F_V = \frac{\left(\frac{D}{\Delta x}\right) SA}{V N_A} \quad \text{(S143)}$$

Here, surface area (SA), volume (V), and diffusion path (Δx) are calculated from crystallographic data of above mentioned MOF's hexagonal pores, with an average diffusion path (Δx) taken as

half the distance needed to enter the pore.[12] Using geometric equations for the surface area and volume of hexagonal prisms, we estimate $F_V \sim 10^{-15}$ s^{-1}.

Next, applying our kinetic framework for a compartmentalized system, we arrive at the following equations for the Negishi reaction operating within MOFs.

$$\gamma = \frac{k_2 k_3 C_{CyZnCl}}{(f_2 + k_2 C_{CyZnCl})(f_3 + k_3)} \tag{S149}$$

$$R_I = \left(\frac{k_1 k_{e2} F_V C_{ArI}}{(f_2 + k_2 C_{CyZnCl})(F_V + k_{e2})}\right)[Pd] \tag{S150}$$

$$TOF = \frac{k_1 k_2 k_3 F_V C_{ArI} C_{CyZnCl}}{(a_1 + F_V)(f_2 + k_2 C_{CyZnCl})(f_3 + k_3)} \tag{S151}$$

The expressions for non-compartmentalized γ', R_I', and TOF' are as follows.

$$\gamma' = \frac{k_2 k_3 C_{CyZnCl}}{(k_2 C_{CyZnCl} + k_{e2})(k_3 + k_{e3})} \tag{S152}$$

$$R_I' = \frac{k_1 k_{e2} [Pd] C_{ArI}}{k_2 C_{CyZnCl} + k_{e2}} \tag{S153}$$

$$TOF' = \frac{k_1 k_2 k_3 C_{ArI} C_{CyZnCl}}{(k_2 C_{CyZnCl} + k_{e2})(k_3 + k_{e3})} \tag{S154}$$

Concentrations were set to 1×10^{-3}, 1×10^{-2}, and 0.1 M for the Pd catalyst, aryl iodied and alkyl zinc chloride substrates respectively, following literature reports.[9,10] Applying $k_1 \sim 10$ M^{-1} s^{-1}, $k_2 \sim 3$ M^{-1} s^{-1}, $k_3 \sim 5$ s^{-1}, and $k_{e2} \sim k_{e3} \sim 2\times10^{-3}$ s^{-1},[10] the Negishi reaction operating within the specified MOF yields $\gamma \sim 1$, $R_I \sim 10^{-19}$ s^{-1} and $TOF \sim 0.2$. The homogeneous analogue is predicted to result in $\gamma \sim 0.98$, $R_I \sim 10^{-6}$ s^{-1} and $TOF \sim 0.5$. This example serves to demonstrate that when a non-compartmentalized reaction already performs at or near $\gamma \sim 1$ and moderate TOF, with deactivation kinetics being much slower than the kinetics of the catalytic cycle, compartmentalization is not necessary and may even worsen catalytic performance.

Table S1. Expressions for compartmentalized and non-compartmentalized key reaction metrics

Physical terms	Scenarios	m[d]	Expression	Equation number
**γ**[a]	Compartmentalized	–	$\frac{k_2 k_3}{(f_2 + k_2)(f_3 + k_3)}$	1, S64
	Non-compartmentalized	–	$\frac{k_2 k_3}{(k_2 + k_{e2})(k_3 + k_{e3})}$	6, S85
**R$_I$**[b]	Compartmentalized	1	$\frac{k_1 k_{e2} F_V^2 C_{Cat} C_A}{(a_1 + F_V)(f_2 + k_2)(F_V + k_{e2})}$	2, S51
		2	$\frac{k_1 k_{e2} F_V C_A}{(f_2 + k_2)(F_V + k_{e2})}\left(\frac{-F_V + \sqrt{F_V^2 + 4a_1 F_V C_{Cat}}}{2a_1}\right)^2$	S53
	Non-compartmentalized	1	$\frac{k_1 k_{e2} C_{Cat} C_A}{k_2 + k_{e2}}$	7, S79
		2	$\frac{k_1 k_{e2} {C_{Cat}}^2 C_A}{k_2 + k_{e2}}$	S79
**TOF**[c]	Compartmentalized	1	$\frac{k_1 k_2 k_3 F_V C_A}{(a_1 + F_V)(f_2 + k_2)(f_3 + k_3)}$	3, S65
		2	$\frac{k_1 k_2 k_3 C_A}{2C_{Cat}(f_2 + k_2)(f_3 + k_3)}\left(\frac{-F_V + \sqrt{F_V^2 + 4a_1 F_V C_{Cat}}}{2a_1}\right)^2$	S66
	Non-compartmentalized	1	$\frac{k_1 k_2 k_3 C_A}{(k_2 + k_{e2})(k_3 + k_{e3})}$	8, S86
		2	$\frac{k_1 k_2 k_3 C_{Cat} C_A}{2(k_2 + k_{e2})(k_3 + k_{e3})}$	S87

[a] γ – reaction efficiency, assesses the ratio of product formation to substrate consumption, [b] R_I – rate of intermediate outflux/elimination, assessment of a compartment's or freely diffusing system's tendency to lose a key intermediate to diffusion to the bulk and/or deactivation, [c] TOF – turnover frequency, product turnovers per unit time normalized to catalyst concentration, [d] order with respect to Cat, denoted as m = 1 or 2, describes first or second order dependence of the oxidative addition step on catalyst concentration. Term a_1 is a consolidation of various kinetic and diffusive parameters used for simplicity (equation S31).

Table S2 Expressions for compartmentalized and non-compartmentalized key reaction metrics for model $C_{Cat,total}$

Physical terms	Scenarios	m[d]	Expression	Equation number
γ[a]	Compartmentalized	–	$\frac{k_2 k_3}{(f_2+k_2)(f_3+k_3)}$	1, S64
	Non-compartmentalized	–	$\frac{k_2 k_3}{(k_2+k_{e2})(k_3+k_{e3})}$	6, S85
R_I[b]	Compartmentalized	1	$\frac{k_1 k_{e2} F_V C_A}{(f_2+k_2)(F_V+k_{e2})}\sqrt{\frac{F_V C_{Cat,total}}{a_2(F_V-a_1)}}$	S122
	Non-compartmentalized	1	$\frac{k_1 k_{e2} C_{Cat,total} C_A}{a_4(k_2+k_{e2})}$	S130
TOF[c]	Compartmentalized	1	$\frac{k_1 k_2 k_3 C_A}{(f_2+k_2)(f_3+f_3)}\sqrt{\frac{F_V}{C_{Cat,total} a_2(F_V-a_1)}}$	S124
	Non-compartmentalized	1	$\frac{k_1 k_2 k_3 C_A}{a_4(k_2+k_{e2})(k_3+k_{e3})}$	S131

This alternative mathematical model, while arithmetically more complex, treats the concentration of all catalytic species more rigorously with minimal changes to mechanistic insights compared to the mathematical model used in the main text (compare Figure 2B–C, 2E–F, 3B–C to Supplemental Figures S5–7). [a] γ – reaction efficiency, assesses the ratio of product formation to substrate consumption, [b] R_I – rate of intermediate outflux/elimination, assessment of a compartment's or freely diffusing system's tendency to lose a key intermediate to diffusion to the bulk and/or deactivation, [c] TOF – turnover frequency, product turnovers per unit time normalized to catalyst concentration, [d] order with respect to Cat, denoted as m = 1 or 2, describes first or second order dependence of the oxidative addition step on catalyst concentration. Terms a_1, a_2, and a_4 are consolidations of various kinetic and diffusive parameters used for simplicity (equations: a_1 – S31, a_2 – S101, a_4 – S128).

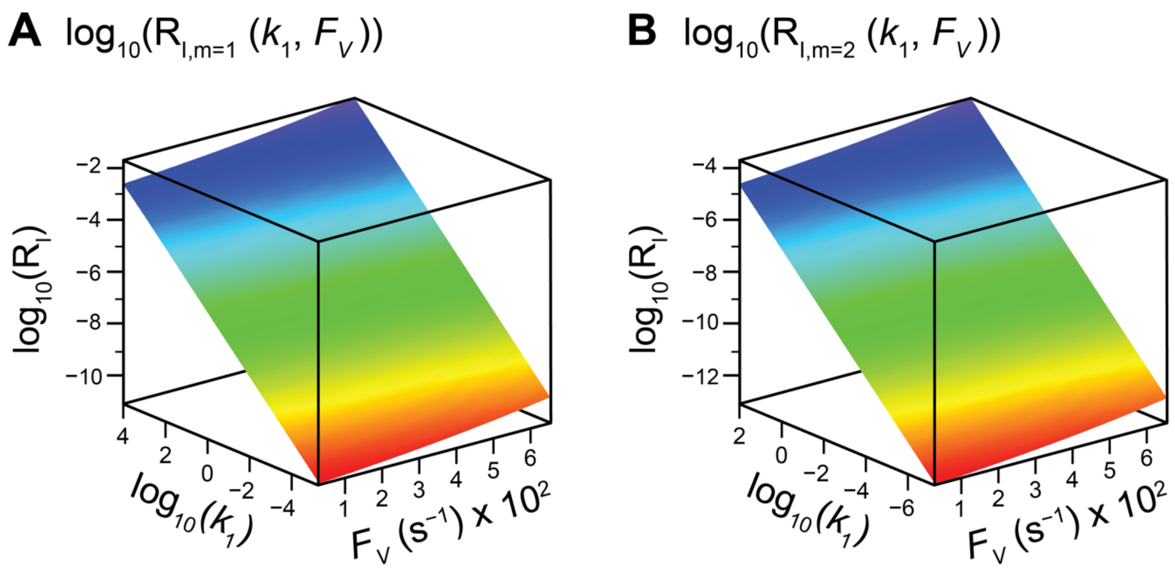

Figure S1. Graphical representations of compartmentalized rate of intermediate outflux (R_I) as a function of volumetric diffusive conductance (F_V) and logarithm of rate constant for oxidative addition (k_1) for (**A**) m = 1 and (**B**) m = 2 both set at rate constants for isomerization/migratory insertion and competing $Cat - A$ elimination, $k_2 = k_{e2} = 1 \times 10^3\ s^{-1}$, and rate constants for reductive elimination and competing $Cat - B$ elimination $k_3 = k_{e3} = 1 \times 10^6\ s^{-1}$.

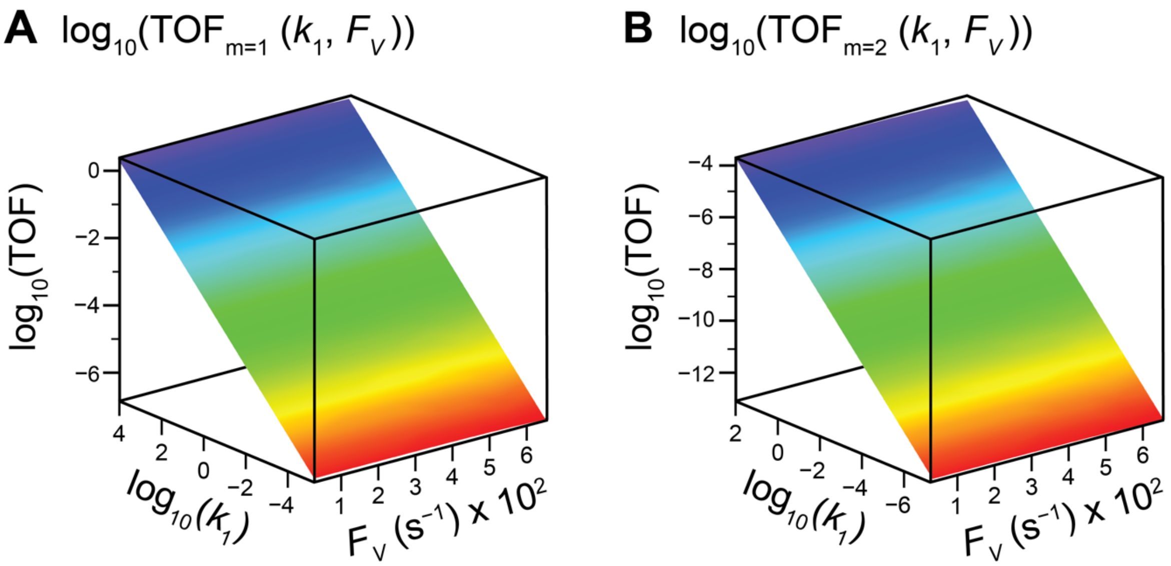

Figure S2. Graphical representations of compartmentalized turnover frequency (TOF) as a function of volumetric diffusive conductance (F_V) and logarithm of rate constant for oxidative addition (k_1) for (**A**) m = 1 and (**B**) m = 2 both set at rate constants for isomerization/migratory insertion and competing $Cat-A$ elimination, k_2 = k_{e2} = 1 × 10^3 s^{-1}, and rate constants for reductive elimination and competing $Cat-B$ elimination k_3 = k_{e3} = 1 × 10^6 s^{-1}.

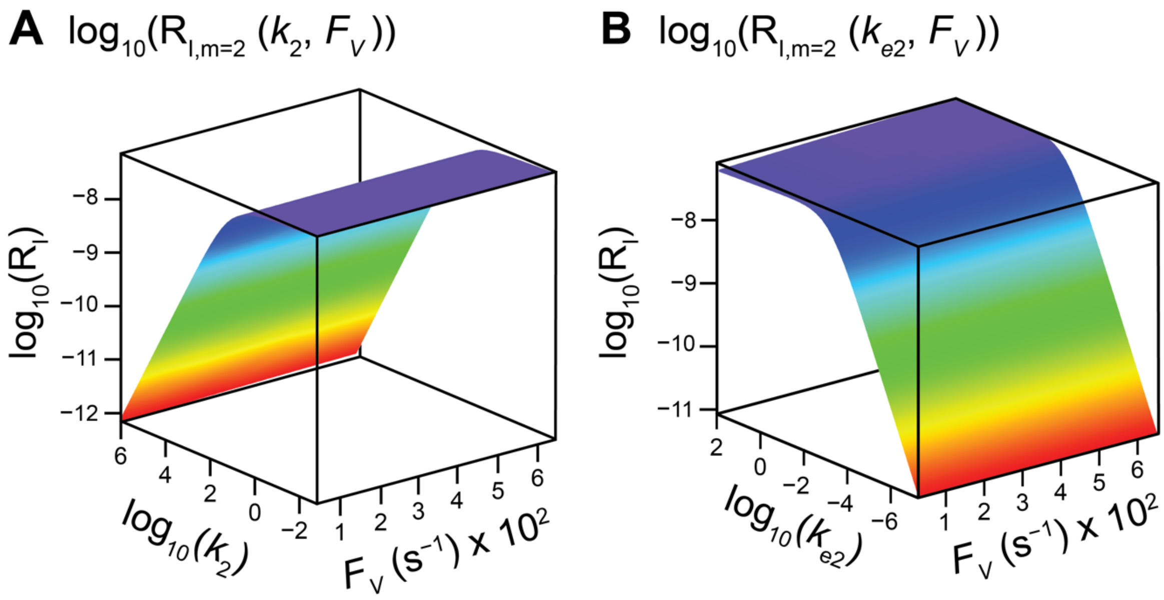

Figure S3. Graphical representations of compartmentalized rate of intermediate outflux (R_I) as a function of volumetric diffusive conductance (F_V) and logarithm of rate constant for isomerization/migratory insertion (k_2) for m = 2 (**A**) at rate constant for oxidative addition k_1 = 10 M^{-2} s^{-1}, rate constant for $Cat - A$ elimination k_{e2} = 1 × 10^3 s^{-1}, and rate constant for reductive elimination and competing $Cat - B$ elimination $k_3 = k_{e3}$ = 1 × 10^6 s^{-1}. (**B**) R_I as a function of F_V and logarithm of rate constant for $Cat - A$ elimination (k_{e2}) for m = 2 at k_1 = 10 M^{-2} s^{-1}, k_2) = 1 × 10^3 s^{-1}, and $k_3 = k_{e3}$ = 1 × 10^6 s^{-1}.

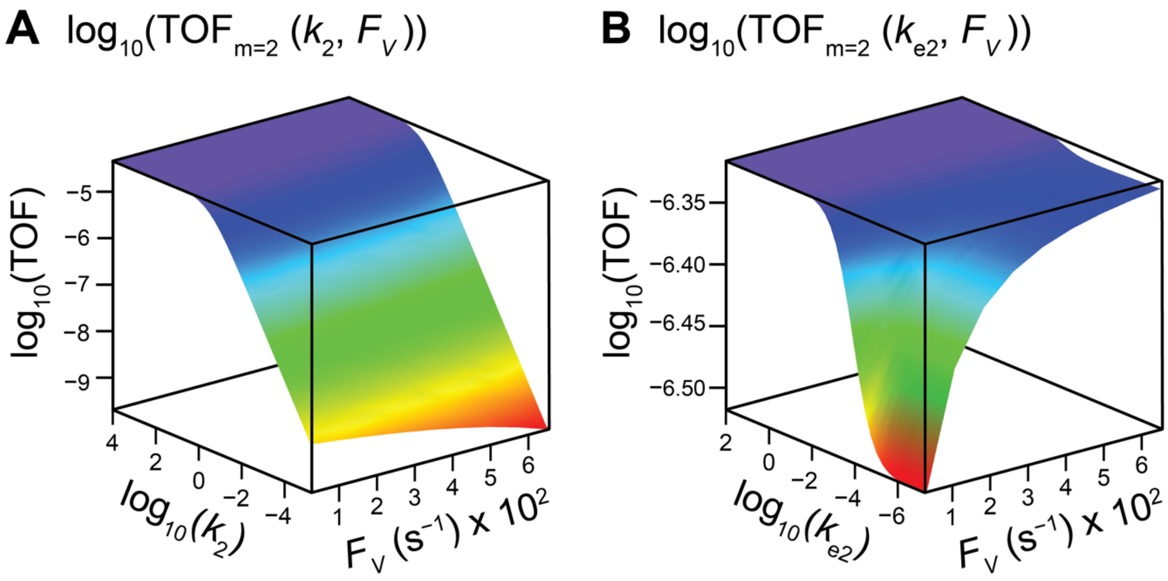

Figure S4. Graphical representations of compartmentalized turnover frequency (TOF) as a function of volumetric diffusive conductance (F_V) and logarithm of rate constant for isomerization/migratory insertion (k_2) for m = 2 (**A**) at rate constant for oxidative addition k_1 = 10 M^{-2} s^{-1}, rate constant for $Cat - A$ elimination k_{e2} = 1 × 10^3 s^{-1}, and rate constant for reductive elimination and competing $Cat - B$ elimination k_3 = k_{e3}= 1 × 10^6 s^{-1}. (**B**) TOF as a function of F_V and logarithm of rate constant for $Cat - A$ elimination (k_{e2}) for m = 2 at k_1= 10 M^{-2} s^{-1}, k_2 = 1 × 10^3 s^{-1}, and k_3 = k_{e3}= 1 × 10^6 s^{-1}.

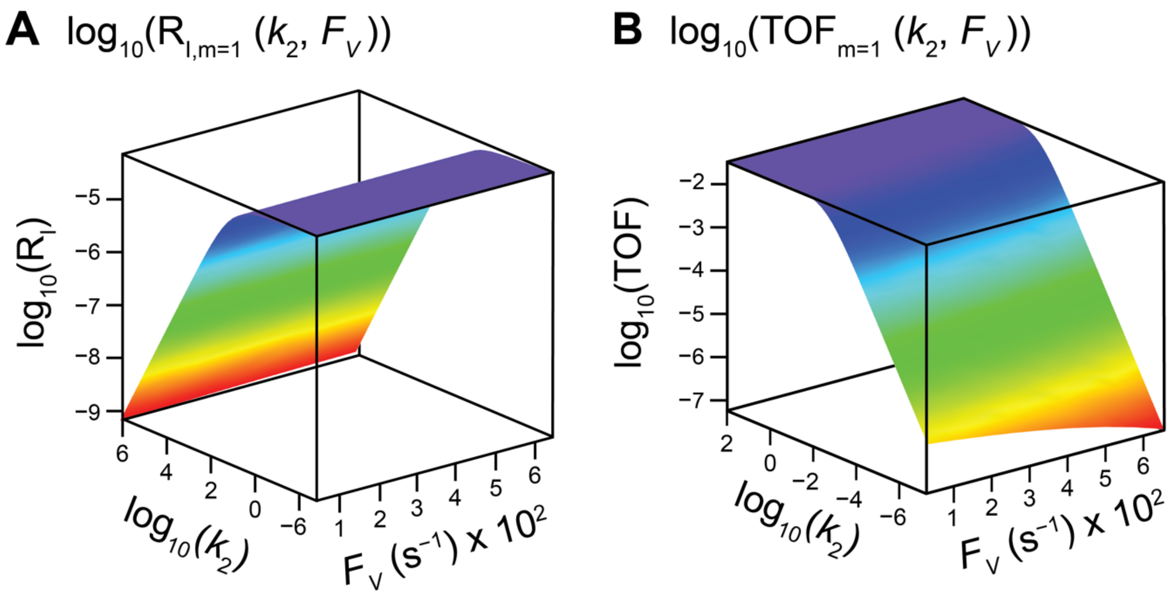

Figure S5. Graphical representations of compartmentalized rate of intermediate outflux (R_I) (**A**) and turnover frequency (TOF) (**B**) under model $C_{Cat,total}$ as a function of volumetric diffusive conductance (F_V) and logarithm of rate constant for isomerization/migratory insertion (k_2) for m = 1. Both panels are set at rate constant for oxidative addition k_1 = 0.1 M^{-1} s^{-1}, rate constant for $Cat - A$ elimination k_{e2} = 1 × 10^{3} s^{-1}, and rate constant for reductive elimination and competing $Cat - B$ elimination $k_3 = k_{e3}$ = 1 × 10^{6} s^{-1}.

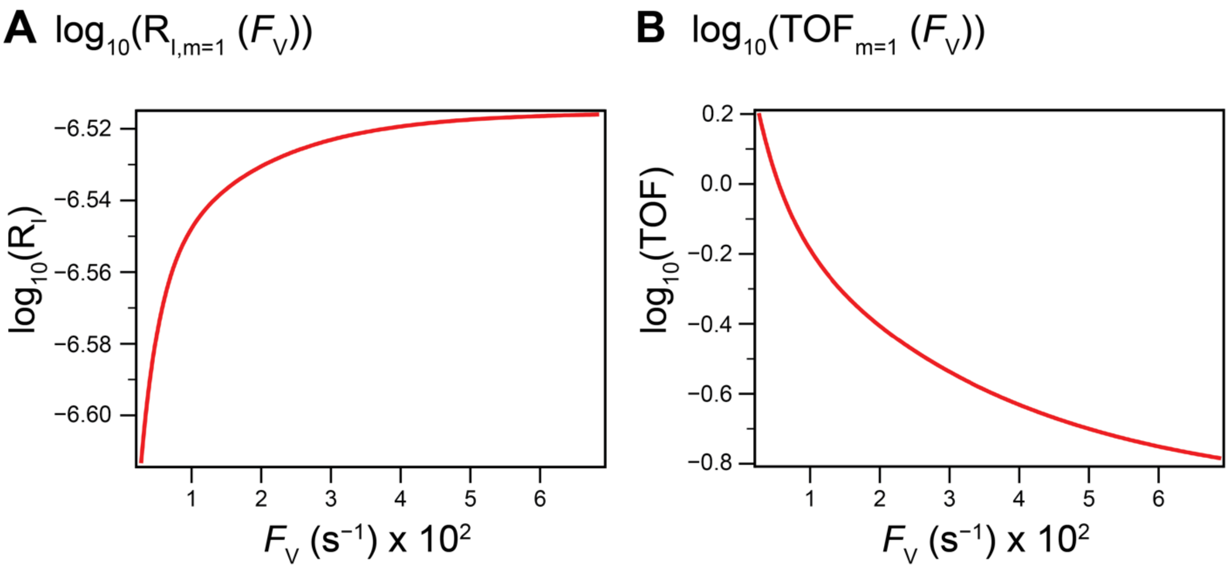

Figure S6. Graphical representations of compartmentalized rate of intermediate outflux (R_I) (**A**) and turnover frequency (TOF) (**B**) under model $C_{Cat,total}$ as a function of volumetric diffusive conductance (F_V) for m = 1. Both panels are set at rate constant for oxidative addition k_1 = 0.1 M^{-1} s^{-1}, rate constant for isomerization/migratory insertion and $Cat - A$ elimination $k_2 = k_{e2}$ = 1 × 10^3 s^{-1}, and rate constant for reductive elimination and competing $Cat - B$ elimination $k_3 = k_{e3}$ = 1 × 10^6 s^{-1}.

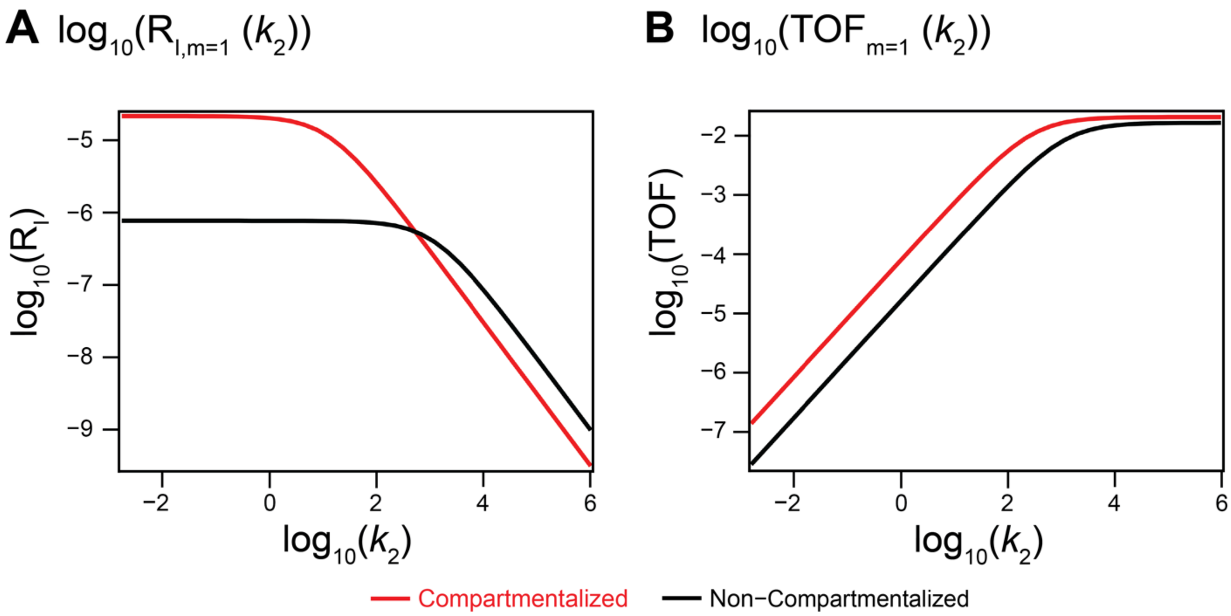

Figure S7. Comparison between compartmentalized and non−compartmentalized rate of intermediate outflux (R_I) (**A**) and turnover frequency (TOF) (**B**) under model $C_{Cat,total}$ as a function of logarithm of rate constant for isomerization/migratory insertion (k_2) for m = 1. Both panels are set at rate constant for oxidative addition k_1 = 0.1 M^{-1} s^{-1}, rate constant for $Cat - A$ elimination k_{e2} = 1 × 10^3 s^{-1}, and rate constant for reductive elimination and competing $Cat - B$ elimination k_3 = k_{e3}= 1 × 10^6 s^{-1}. The compartmentalized trace in both panels are set to volumetric diffusive conductance (F_V) of 320 s^{-1}.

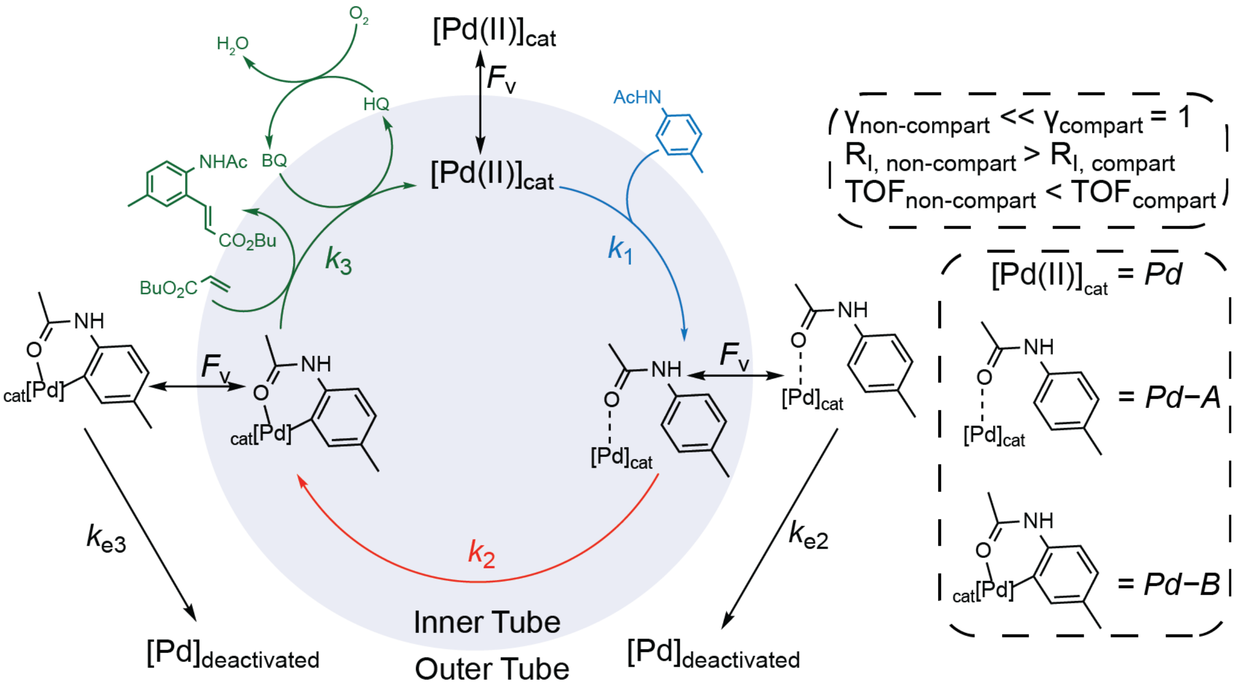

Figure S8. Schematic of compartmentalized palladium catalyzed olefin arylation (Fujiwara-Mirotani reaction)[5–7] operating in a tube-in-tube reactor[8] as discussed in Supplementary Section 4. F_V, volumetric diffusive conductance; k_1, k_2, and k_3, rate constants for catalytic steps of the Fujiwara-Mirotani reaction; k_{e2} and k_{e3}, rate constants for deactivation of palladium species by oxidizing media; BQ, benzoquinone; HQ, hydroquinone; γ, reaction efficiency; R_I, intermediate outflux and subsequent elimination; TOF, turnover frequency; compart, compartmentalized; non-compart, non-compartmentalized. Terms Pd, $Pd - A$, and $Pd - B$ are abbreviations used in Supplemental Section 4.

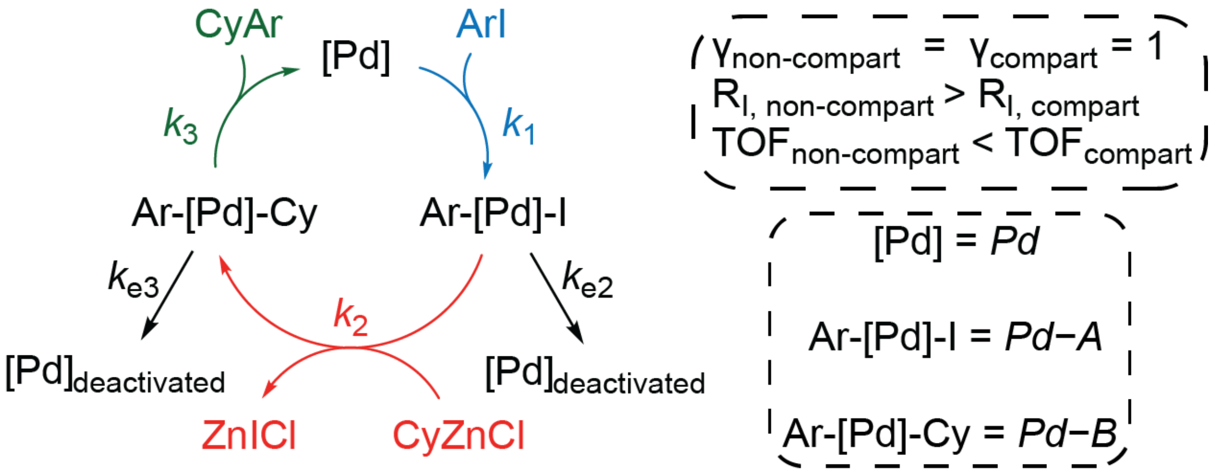

Figure S9. Schematic of palladium catalyzed olefin arylation (Negishi coupling reaction)[9,10] used in Supplementary Section 5. F_V, volumetric diffusive conductance; k_1, k_2, and k_3, rate constants for catalytic steps of the Negishi reaction; k_{e2} and k_{e3}, rate constants for deactivation of palladium species. Ar, aryl; Cy, cyclohexyl; γ, reaction efficiency; R_I, intermediate outflux and subsequent elimination; TOF , turnover frequency; compart, compartmentalized; non-compart, non-compartmentalized. Terms Pd, $Pd - A$, and $Pd - B$ are abbreviations used in Supplemental Section 5.

Additional references

1. S. Tsitkov and H. Hess, *ACS Catal.*, 2019, **9**, 2432−2439.
2. B. S. Natinsky, B. J. Jolly, D. M. Dumas and C. Liu, *Chem. Sci.*, 2021, **12**, 1818−1825.
3. W. Stein, *Transport and diffusion across cell membranes*, Elsevier Inc., 1986.
4. B. S. Natinsky, S. Lu, E. D. Copeland, J. C. Quintana and C. Liu, *ACS Cent. Sci.*, 2019, **5**, 1584−1590.
5. I. Moritani and Y. Fujiwara, *Tetrahedron Lett.*, 1967, **8**, 1119−1122.
6. Y. Fujiwara, I. Moritani, S. Danno, R. Asano, and S. Teranishi, *J. Am. Chem. Soc.*, 1969, **91**, 7166−7169.
7. C. J. Mulligan, J. S. Parker, and K. K. Hii, *React. Chem. Eng.*, 2020, **5**, 1104−1111.
8. F. Ferlin, I. Anastasiou, L. Carpisassi, and L. Vaccaro, *Green Chem.*, 2021, **23**, 6576−6582.
9. E. Negishi, A. O. King, and N. Okukado, *J. Org. Chem.*, 1977, **42**, 1821−1823.
10. H. Zhang, X. Luo, K. Wongkhan, H. Duan, Q. Li, L. Zhu, J. Wang, A. S. Batsanov, J. A. Howard, and T. B. Marder, *Chem. Eur. J.*, 2009, **15**, 3823−3829.
11. E. Tiburcio, R. Greco, M. Mon, J. Ballesteros-Soberanas, J. s. Ferrando-Soria, M. López-Haro, J. C. Hernández-Garrido, J. Oliver-Meseguer, C. Marini and M. Boronat, *J. Am. Chem. Soc.*, 2021, **143**, 2581−2592.
12. V. P. Ananikov, S. S. Zalesskiy, V. V. Kachala and I. P. Beletskaya, *J. Organomet. Chem.*, 2011, **696**, 400−405.
